# Checklist-Guided Code Status Discussions in Patients for Whom Cardiopulmonary Resuscitation Is Considered Futile: An Analysis of a Randomized Clinical Trial

**DOI:** 10.1001/jamanetworkopen.2025.33638

**Published:** 2025-09-25

**Authors:** Armon Arpagaus, Leta Arpagaus, Christoph Becker, Sebastian Gross, Flavio Gössi, Benjamin Bissmann, Samuel Kaspar Zumbrunn, Philipp Schuetz, Jörg D. Leuppi, Drahomir Aujesky, Balthasar Hug, Thomas Peters, Stefano Bassetti, Sabina Hunziker

**Affiliations:** 1 Division of Medical Communication/Psychosomatic Medicine, University Hospital Basel, Basel, Switzerland; 2 Division of Internal Medicine, University Hospital Basel, Basel, Switzerland; 3 Medical Outpatient Department, University Hospital Basel, Basel, Switzerland; 4 Faculty of Medicine, University of Basel, Basel, Switzerland; 5 Division of Internal Medicine, Cantonal Hospital Aarau, Aarau, Switzerland; 6 University Center of Internal Medicine, Cantonal Hospital Baselland, Liestal, Switzerland; 7 Department of General Internal Medicine, Inselspital, Bern University Hospital, University of Bern, Bern, Switzerland; 8 Division of Internal Medicine, Cantonal Hospital Lucerne, Lucerne, Switzerland; 9 Faculty of Health Sciences and Medicine, University of Lucerne, Lucerne, Switzerland; 10 Division of Internal Medicine, St Claraspital AG, Basel, Switzerland; 11 Department of Psychosomatic Medicine, University Hospital Basel, Basel, Switzerland

## Abstract

**Importance:**

Code status discussions represent a fundamental aspect of advanced care planning and impose major challenges for clinicians in patients for whom cardiopulmonary resuscitation (CPR) is considered futile.

**Objective:**

To investigate the effect of a structured communication approach in code status discussions on decisions regarding code status and various quality-of-care measures for patients in whom resuscitation is considered futile.

**Design, Setting, and Participants:**

The GUIDE trial is a multicenter randomized clinical trial, which included patients deemed futile regarding CPR measures. The study was conducted between June 1, 2019, and April 30, 2023, in medical inpatients wards across 6 Swiss teaching hospitals. Medical inpatients for whom CPR measures were considered futile based on a prearrest Good Outcome Following Attempted Resuscitation score of 14 or higher or a Clinical Frailty Scale score of 7 or higher were eligible. Patients with cognitive or physical condition hindering meaningful conversation were excluded.

**Main Outcomes and Measures:**

Main outcomes included patients’ code status decisions as well as preference for mechanical ventilatory assistance and intensive care unit admission. Additional outcomes assessed patients’ psychological burden after the discussion measured by the State-Trait Anxiety Inventory and Hospital Anxiety and Depression scale and physicians’ perception regarding the discussions.

**Results:**

A total of 177 patients (mean [SD] age, 76.3 [12.0] years; 90 [51%] female) were studied. Overall, the rate of do-not-resuscitate orders was 85%. No significant difference was observed between groups (checklist vs usual care group, 79 of 89 [89%] vs 72 of 88 [82%]; odds ratio, 1.76; 95% CI, 0.75-4.12; *P* = .20). However, patients in the checklist group were less likely to prefer intensive care unit admission compared with usual care (31 of 89 [36%] vs 44 of 88 [52%]; odds ratio, 0.53; 95% CI, 0.29-0.99; *P* = .046). Physicians perceived code status discussions using the checklist less challenging (mean [SD], 3.5 [2.8] vs 4.7 [2.8]; difference, −1.23; 95% CI, −2.1 to −0.35; *P* = .006). There was no significant difference in patients’ psychological reaction to code status discussions measured by State-Trait Anxiety Inventory and Hospital Anxiety and Depression scale.

**Conclusions and Relevance:**

This analysis of a randomized clinical trial found that checklist-guided code status discussions in patients for whom CPR was considered futile reduced their preference for intensive care unit admission while alleviating physicians’ challenges during code status discussions without adversely affecting patients’ psychosocial burden.

**Trial Registration:**

ClinicalTrials.gov Identifier: NCT03872154

## Key Points

**Question:**

Does a structured communication approach have an effect on code status discussions in patients in whom an attempt at cardiopulmonary resuscitation (CPR) is considered futile?

**Findings:**

In this randomized clinical trial of 177 patients in whom CPR is deemed futile, a checklist-guided code status discussion had no effect on code status decisions but significantly reduced patients’ preference for intensive care treatment, and physicians reported these discussions as less challenging.

**Meaning:**

These results suggest that a structured communication approach may improve quality of code status discussions in patients for whom CPR attempts are considered futile.

## Teaser

This randomized clinical trial of patients with cardiopulmonary resuscitation (CPR) futility examines whether a structured, checklist-guided approach to code status discussions can enhance communication and increase the quality of care.

## Introduction

Code status discussions represent a critical component of medical care, particularly for patients facing severe illness or nearing the end of life.^1^ These conversations are complex, requiring clinicians to balance medical realities with patient values and preferences while addressing emotionally charged topics.^2^ In patients for whom cardiopulmonary resuscitation (CPR) is considered futile, prognosis after in-hospital cardiac arrest worsens further with advanced illness or major comorbidities, such as metastatic cancer or end-stage heart or lung disease.^3^ These patients have extremely low survival rates and often poor neurologic outcomes, complicating discussions about CPR appropriateness.^4,5,6,7^

In medicine, futility describes situations where interventions or therapies are unlikely to achieve meaningful benefits, such as improved survival or quality of life. Such therapies are deemed clinically inappropriate when potential harm exceeds anticipated benefit based on clinical evidence and professional judgment. In CPR, futility specifically arises when the likelihood of achieving meaningful survival is extremely low. Such circumstances often pose complex ethical and clinical dilemmas, further complicated by the lack of a universally accepted definition of CPR futility.^8,9^ Although some define CPR futility as situations in which survival is highly improbable or where the potential harm of resuscitation outweighs the benefit, others argue its subjectivity.^10,11,12,13^ To address this, objective scoring tools, such as the prearrest Good Outcome Following Attempted Resuscitation (GO-FAR) score and the Clinical Frailty Scale (CFS), have been developed and assessed.^14,15,16^ These tools have demonstrated strong prognostic accuracy in identifying patients for whom CPR is unlikely to provide meaningful benefit.^17,18^ For example, the GO-FAR score predicts neurologically intact survival after in-hospital cardiac arrest and has been externally validated in several countries.^19,20,21,22^ The CFS quantifies frailty severity and has been associated with poor overall and neurologic outcomes after CPR.^23,24^ These tools provide a framework for guiding decisions regarding CPR futility, offering an opportunity to improve the transparency and consistency of code status discussions.

Despite their utility, few studies^25,26^ address communication strategies specifically tailored to patients for whom CPR is considered futile. Evidence suggests that structured discussions—grounded in clear communication of prognosis and shared decision-making—can enhance the quality of care and improve alignment with patient goals. However, in the specific context where CPR is considered futile, physicians may face challenges in balancing the delivery of poor prognostic information with the need to maintain patient trust and minimize psychological distress. Research on discussing code status in this at-risk population remains sparse. This study hypothesizes that a structured, checklist-guided approach to code status discussions can enhance the quality of communication, support physicians in guiding these difficult conversations, and increase the quality of care in patients for whom CPR is considered futile.

## Methods

### Design, Setting, and Population

The GUIDE trial is a multicenter cluster randomized trial conducted in the internal medicine divisions of 6 Swiss teaching hospitals between June 1, 2019, and April 30, 2023. The GUIDE trial was conducted alongside the CLEAR trial and used the same fundamental screening criteria.^27^ However, it exclusively included patients considered futile for CPR and therefore ineligible for participation in the CLEAR trial. For this purpose, newly admitted patients were evaluated by the study team, and their expected CPR outcomes were assessed using the GO-FAR score and the CFS (eMethods 1 in Supplement 1). Patients with acceptable probabilities (survival with good neurologic outcome ≥1.7%) were included in the main CLEAR trial, which used a shared decision-making checklist. Patients for whom resuscitation was deemed futile, defined by a GO-FAR score of 14 or higher (indicating a chance of survival with minimal neurological disability <1.7%^13,15^) or CFS score of 7 or higher (indicating severe frailty with debilitating conditions^16^), were included in the GUIDE trial, reported in this article. Race and ethnicity data were not collected because this information is not routinely recorded in Swiss medical records. Of 472 patients screened, 295 met exclusion criteria and were not studied. Ethical approval was obtained by the local ethics committee of Northwestern and Central Switzerland for the GUIDE and CLEAR trials. At each hospital, internal medicine department heads permitted residents to conduct code status discussions using a predefined checklist as part of routine care. Written informed consent was acquired from patients for data collection and follow-up. The trial was registered on ClinicalTrials.gov before its start on March 13, 2019. The trial was reported in accordance with the Consolidated Standards of Reporting Trials (CONSORT) reporting guidelines.^28^

### Study Randomization

Residents on medical wards were randomly assigned to either training for a code status discussion using a checklist (checklist group) or general patient-centered communication training (usual care group). Block randomization stratified by hospital was performed (block size of 4-6) within each stratum using a data collection system (SecuTrial, interActive Systems GmbH). All residents conducted systematic code status discussion with consecutive adult patients admitted to the ward during 3 weeks. Patients were eligible regardless of primary diagnosis, except those with cognitive or physical impairments (eg, dementia and delirium), severe mental illness, or significant hearing loss that hindered meaningful discussions.

Responsible residents were notified if the patient met the criteria for CPR futility, regardless of study arm. In the checklist group, residents used the predefined GUIDE checklist for discussing code status with patients for whom CPR was deemed futile. Blinding of patients and physicians was ensured by requesting participation in a trial comparing 2 communication strategies for code status discussions, without revealing intervention details before randomization.

### Study Flow and Description of Intervention

**Figure 1.**
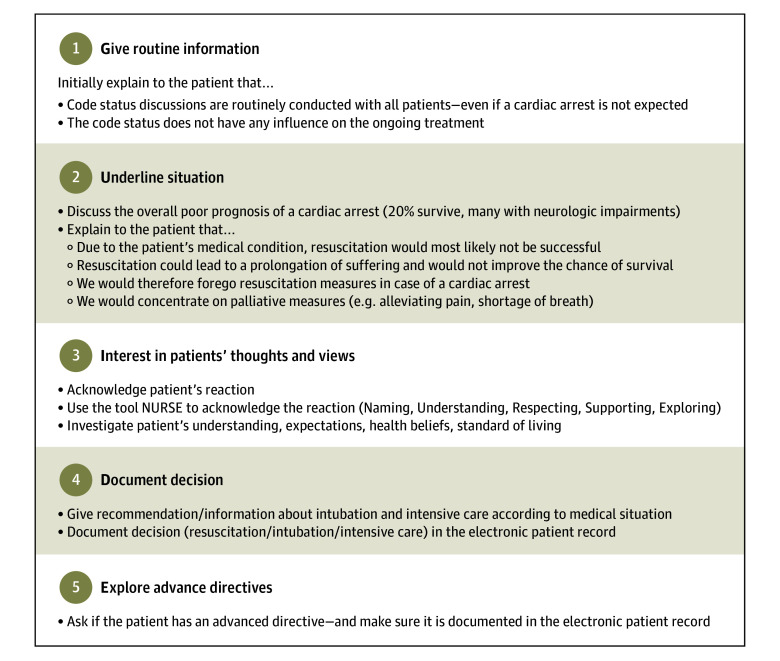
GUIDE: Checklist for Code Status Discussion in Patients With Cardiopulmonary Resuscitation (CPR) Futility The GUIDE checklist was used for code status discussions in the intervention group in patients for whom CPR measures were deemed futile according to a Good Outcome Following Attempted Resuscitation (GO-FAR) score of 14 points or higher or a Clinical Frailty Scale (CFS) of 7 points or higher. Each of the 5 steps is reflected in 1 letter of the GUIDE acronym.

Residents in the intervention group attended a 1-hour communication workshop led by research team members experienced in teaching medical communication. During the workshop for the main study, residents received training on a distinct checklist for patients with CPR futility. This training provided structured guidance for code status discussions with patients in whom CPR was considered futile, using a checklist to facilitate patient-centered communication about prognosis, likely outcomes of resuscitation, and alternative care approaches (Figure 1). The key component of the checklist was the information that the medical team would forgo resuscitation measures in case of cardiac arrest and focus on palliative measures. The training included a simulated code status conversation using the checklist, followed by feedback from the workshop instructor.

The checklist was developed based on clinical expertise, contributions from patients and health care professionals, and insights from existing literature.^1,25,26,29^ It was structured in 5 categories, under the acronym GUIDE, which represents 5 key steps: give routine information, underline situation, interest in patients’ thoughts and views, document decision, and explore advance directives (eMethods 2 in Supplement 1).

Code status discussions were conducted upon patient admission. To ensure adherence, the workshop instructor joined at least 3 discussions per resident, providing feedback and, when necessary, rehearsal of specific training components. To reduce bias, residents randomized in the usual care group attended a similarly timed workshop covering the importance of code status discussions and general communication skills, focusing on structuring information and responding to emotions, without a checklist.

### Patient Involvement in Trial Design

A stakeholder advisory group consisting of 25 patients hospitalized in the medical wards of the University Hospital of Basel and 15 clinicians (physicians and nurses) experienced in end-of-life, emergency, or intensive care was involved in designing the study and intervention and reviewing the study protocol (Supplement 2) according to patient and public involvement strategies. The advisory group assisted in prioritizing and defining outcomes and provided input on the intervention, particularly regarding the checklist, which was further refined with input from additional clinical experts.

### Data Collection

Data were collected at 3 time points by team members blinded toward the allocation arm (ie, at baseline, within 24 hours after the discussion, and 30 days after hospital discharge). Baseline data included patients’ sociodemographics, medical conditions, comorbidities, and data necessary for a calculation of the Charlson Comorbidity Index^30^ and the National Early Warning Score 2.^31^ After the code status discussion, a research team member interviewed patients to assess secondary end points.

### Outcome Measures

The primary outcome was the documented code status in the electronic patient record on the day of code status discussions (rate of do-not-resuscitate [DNR] orders and preferences for mechanical ventilatory support and intensive care unit [ICU] admission). The key secondary outcomes were patients’ psychological burden assessed by validated German translations of the Hospital Anxiety and Depression Scale (HADS)^32^ and the State-Trait Anxiety Inventory (STAI).^33^ Both are validated tools for assessing anxiety and depression symptoms in various medical settings and reliably show short-term fluctuations.^34,35,36^

Furthermore, we assessed patients’ concerns and fears using a visual analogue scale of 0 to 10 to capture patient-reported worries about resuscitation, cardiac arrest, and perceived pressure during discussions, with 0 indicating low and 10 indicating high perceived pressure. Physicians’ perceptions were also evaluated. Physicians rated discussion difficulty and satisfaction using a visual analogue scale of 0 to 10, with 0 indicating low and 10 indicating high perceived pressure. Additional outcomes, including ICU admissions, length of hospital stay, readmissions, and 30-day mortality, were extracted from medical records (eMethods 3 in Supplement 1).

### Statistical Analysis

Baseline characteristics and further factors were stratified according to the randomization group. The Pearson χ^2^ test was used for testing differences between categorical variables and 2-tailed, unpaired *t* tests for continuous outcomes as appropriate after evaluation for normality. *P* < .05 was considered statistically significant. Additionally, univariable logistic or linear regression analysis was used to evaluate for associations of potential factors with outcomes. Missing data and their corresponding reasons are given in eTables 2 to 5 in Supplement 1. Missing data were addressed using multiple imputation in the HADS and STAI scores according to their manuals (eMethods 3 in Supplement 1). All statistical analyses were performed using Stata software, version 15.0 (StataCorp).

## Results

### Study Flow and Baseline Characteristics

**Figure 2.**
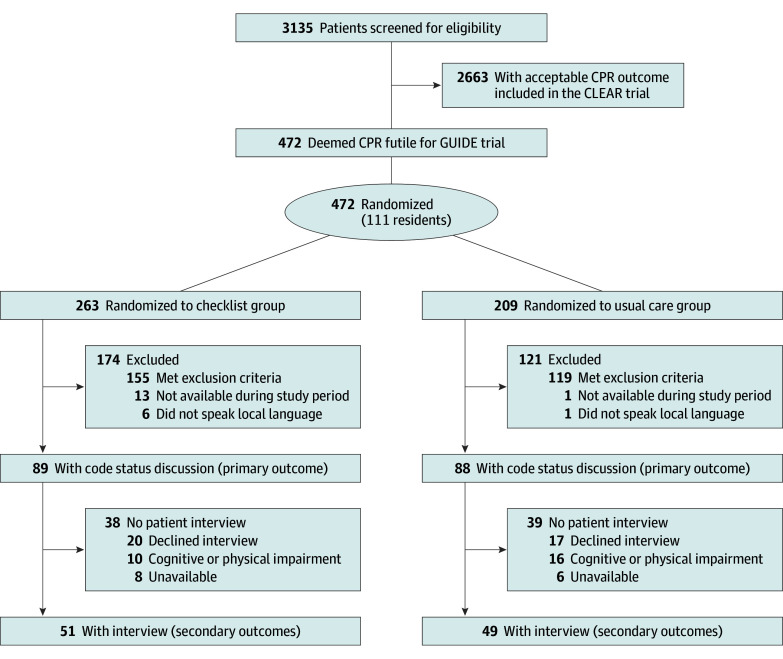
Flowchart of the Study Patients Flowchart of the GUIDE trial, which included patients alongside the CLEAR trial. Patients in whom cardiopulmonary resuscitation (CPR) was deemed futile (Good Outcome Following Attempted Resuscitation score ≥14 points or a Clinical Frailty Scale score ≥7 points) were eligible for inclusion in the GUIDE trial. GUIDE: Checklist-Guided Code Status Discussions in Patients for Whom Cardiopulmonary Resuscitation Is Considered Futile; acronym for a communication checklist consisting of 5 steps: Give routine information, Underline situation, Interest in patients’ thoughts and views, Document decision, Explore advance directives. CLEAR: Shared Decision-Making in Code Status Discussions in Patients with Acceptable Outcomes; acronym for a communication checklist consisting of 5 steps: Clinician-patient engagement, Learn and inform, Explore patient preferences, Assess and document, Review advance directives.

A total of 111 residents caring for patients for whom CPR was considered futile were included. A total of 177 patients (mean [SD] age, 76.3 [12.0] years; 90 [51%] female and 87 [49%] male) were included in the analysis (eTable 1 in Supplement 1), including 89 patients randomized to the checklist group and 88 in the usual care group (Figure 2). A large proportion of the patients had multiple comorbidities, reflected by a mean (SD) Charlson Comorbidity Index of 7.6 (2.6) points. Primary admission diagnoses were cancer (50 [28%]), infectious diseases (46 [26%]), and cardiologic diseases (20 [11%]). The randomization arms were well balanced in terms of admission diagnoses, comorbidities, and other clinical parameters (Table 1).

**Table 1.** Baseline Characteristics of the Study Participants

Characteristic	No. (%) of participants^a^	
	Usual care group (88 patients and 55 residents)	Checklist group (89 patients and 56 residents)
**Patients**		
Age, mean (SD), y	76.7 (12.0)	76.0 (12.0)
Sex		
Male	45 (51)	42 (47)
Female	43 (49)	47 (53)
Civil status		
Single	13 (15)	13 (15)
Married or in a relationship	40 (47)	42 (48)
Divorced	7 (8)	7 (8)
Widowed	26 (30)	26 (30)
Having children (yes)	66 (77)	61 (69)
No. of children, mean (SD)	1.8 (0.9)	2.0 (0.9)
Citizenship		
Switzerland	71 (81)	70 (79)
Germany	5 (6)	4 (4)
Italy	4 (5)	7 (8)
Other^b^	8 (9)	8 (9)
Religious affiliation		
None	20 (23)	16 (18)
Christian	62 (71)	65 (74)
Other^c^	5 (6)	7 (8)
Principal diagnoses on admission		
Circulatory diseases	8 (9)	12 (13)
Cancer	23 (26)	27 (30)
Infectious or respiratory diseases	28 (32)	18 (20)
Gastroenterology or metabolism	7 (8)	11 (12)
Neurologic diseases	8 (9)	4 (4)
Other^d^	14 (16)	17 (19)
Comorbidities		
Circulatory diseases	42 (48)	45 (51)
Neurologic diseases	36 (41)	38 (43)
Cancer	39 (44)	35 (39)
Gastroenterologic diseases	23 (26)	28 (31)
Metabolic diseases	25 (28)	38 (43)
Respiratory diseases	29 (33)	26 (29)
Infectious diseases	9 (10)	10 (11)
Nephrologic diseases	33 (38)	29 (33)
Rheumatologic diseases	7 (8)	9 (10)
Psychiatric diseases (excluding depression)	8 (9)	6 (7)
Depression	7 (8)	2 (2)
Other comorbidities^e^	45 (51)	50 (56)
Charlson Comorbidity Index score, mean (SD)	7.3 (2.6)	7.8 (2.3)
Quality of life at baseline (EQ-5D-3L)		
EQ-5D-3L Index score,^f^ mean (SD)	0.5 (0.4)	0.5 (0.4)
EQ-5D-3L VAS score,^g^ mean (SD)	48.4 (23.5)	49.3 (20.6)
Clinical values		
NEWS2 score, mean (SD)	3.5 (3.2)	2.9 (2.5)
GO-FAR score, mean (SD)	12.9 (7.5)	12.6 (8.2)
Survival according to GO-FAR score		
0.9% Good neurologic outcome	5 (6)	5 (6)
1.7% Good neurologic outcome	53 (60)	48 (54)
9.4% Good neurologic outcome	30 (34)	36 (40)
Clinical Frailty Scale score, mean (SD)	6.6 (1.6)	7.0 (1.6)
**Residents**		
Age, mean (SD), y	31.3 (3.6)	30.4 (2.8)
Sex		
Male	38 (51)	27 (48)
Female	27 (49)	29 (52)
Primary language		
German	45 (82)	46 (82)
Non-German	10 (18)	10 (18)
Job experience, mean (SD), y	3.9 (2.4)	3.7 (2.1)

Abbreviations: EQ-5D-3L, European Quality of Life 5 Dimensions; GO-FAR, Good Outcome Following Attempted Resuscitation score; NEWS2, National Early Warning score 2; VAS, visual analogue scale.

^a^ Unless otherwise indicated.

^b^ Other citizenships included Turkey, Portugal, Macedonia, Serbia, Spain, and Albania.

^c^ Including Jewish, Muslim, and Hindu.

^d^ Other principal diagnosis at admission included nephrologic diseases, rheumatologic diseases, psychiatric diseases, urologic diseases, benign hematologic diseases (ie, anemia), insomnia, orthopedic diseases, otorhinolaryngologic diseases, ophthalmologic diseases, and cachexia.

^e^ Other comorbidities included urologic diseases, benign hematologic diseases (ie, anemia), insomnia, orthopedic diseases, otorhinolaryngologic diseases, ophthalmologic diseases, and cachexia.

^f^ EQ-5D-3L Index scores range from −0.205 to 1, with higher scores indicating better quality of life.

^g^ EQ-5D-3L VAS scores range from 0 to 100, with higher scores indicating higher self-reported health.

### Primary Outcome: Rate of DNR Orders

Overall, the rate of DNR orders was 85%. No significant difference was observed between groups (79 of 89 [89%] for the checklist group vs 72 of 88 [82%] for the usual care group; odds ratio [OR], 1.76; 95% CI, 0.75-4.12; *P* = .20) (Table 2).

**Table 2.** Primary and Secondary End Points

End point	Total No.	Usual care group (n = 88)	Checklist group (n = 89)	*P* value	Difference (95% CI)	*P* value
Ultimate code status from medical record						
Documented DNR code status, No. (%)	177	72 (82)	79 (89)	.19	OR, 1.76 (95% CI, 0.75 to 4.12)	.20
Documented preference for mechanical ventilatory support, No. (%)	154	23 (31)	19 (24)	.31	OR, 0.69 (95% CI, 0.34 to 1.41)	.31
Documented preference for ICU admission, No. (%)	170	44 (52)	31 (36)	.045^a^	OR, 0.53 (95% CI, 0.29 to 0.99)	.046^a^
Patient-reported outcomes						
Patients’ concerns and fears						
Patients’ disturbance caused by the discussion (VAS of 0-10), mean (SD)^a^	81	1.3 (2.2)	1.2 (2.3)	.82	−0.12 (−1.13 to 0.89)	.82
Patients’ fear of pain from an actual cardiac arrest (VAS of 0-10), mean (SD)^a^	80	0.4 (1.4)	0.7 (1.9)	.53	0.24 (−0.52 to 1.00)	.53
Patients’ fear of pain from a life-threatening disease (VAS of 0-10), mean (SD)^a^	80	0.5 (1.4)	0.9 (2.2)	.38	0.37 (−0.47 to 1.21)	.38
Patients’ perceived feeling of being put under pressure during discussion (VAS of 0-10), mean (SD)^a^	79	0.4 (1.4)	0.4 (1.3)	.94	−0.02 (−0.62 to 0.58)	.94
Patients’ reported psychological burden						
State Trait Anxiety Inventory score, mean (SD)	77	39.1 (13.0)	39.8 (10.9)	.79	0.70 (−4.72 to 6.15)	.79
Hospital Anxiety and Depression Scale–anxiety over cutoff of 8, No. (%)	75	32 (86)	32 (86)	.78	OR, 1.20 (95% CI, 0.33 to 4.33)	.78
Hospital Anxiety and Depression Scale–depression over cutoff of 8, No. (%)	73	26 (70)	25 (69)	.94	OR, 0.96 (95% CI, 0.35 to 2.61)	.94
Hospital Anxiety and Depression Scale total score, mean (SD)	73	19.5 (4.3)	19.1 (3.8)	.65	−0.43 (−2.32 to 1.46)	.65
Physicians’ perception of resuscitations discussion						
Physicians’ overall satisfaction with resuscitation discussion (VAS score, 0-10), mean (SD)^a^	159	7.0 (2.2)	7.5 (2.0)	.08	0.58 (−0.08 to 1.23)	.08
Physicians’ perceived time management of the discussion (VAS of 0-10), mean (SD)^a^	159	7.2 (2.3)	7.6 (1.9)	.19	0.44 (−0.22 to 1.09)	.18
Physicians’ perceived difficulty of the discussion (VAS of 0-10), mean (SD)^a^	159	4.7 (2.8)	3.5 (2.8)	.006	−1.23 (−2.10 to −0.35)	.006
30-d Outcomes						
Length of hospital stay, mean (SD), d	177	12.3 (9.7)	11.0 (9.5)	.38	−1.26 (−4.11 to 1.59)	.38
ICU admission after resuscitation discussion, No. (%)	177	5 (6)	6 (7)	.77	OR, 1.20 (95% CI, 0.35 to 4.09)	.77
Readmission to hospital within 30 d, No. (%)	177	15 (17)	13 (15)	.66	OR, 0.83 (95% CI, 0.37 to 1.87)	.66
Death within 30 d, No. (%)	177	23 (26)	24 (27)	.90	OR, 1.04 (95% CI, 0.54 to 2.03)	.90

Abbreviations: DNR, do-not-resuscitate; ICU, intensive care unit; OR, odds ratio; VAS, visual analogue scale.

^a^ VAS scores range from 0 to 10, with higher scores indicating a greater perceived level of the reported factor.

### Secondary Outcomes

#### Preferences Regarding Mechanical Ventilatory Support and ICU Admission

Patients in the checklist group had a significantly lower preference of admission to ICU (31 of 85 [36%] vs 44 of 85 [52%]; OR, 0.53; 95% CI, 0.29-0.99; *P* = .046) compared with the usual care group. No differences in preference were found for mechanical ventilatory support in case of clinical deterioration (19 of 80 [24%] vs 23 of 74 [31%]; OR, 0.69; 95% CI, 0.34-1.41 *P* = .31).

#### Patient-Reported Outcomes

Of the 177 patients, 100 were available for further interviews (Figure 2). Overall, no significant differences were observed between groups regarding patients’ fears, concerns, or psychological burden: scores for disturbance caused by the discussion, fear of pain from an actual cardiac arrest, fear of pain from a life-threatening disease, and feeling of being put under pressure during the discussion showed similar scores in both groups (Table 2). In addition, there was no difference between the checklist and the usual care group in the further assessment of the psychological burden, with comparable STAI and HADS scores showing no significant difference.

#### Physician-Associated Outcomes and Outcomes at 30-Day Follow-Up

Residents in the checklist group reported lower difficulty conducting code status discussions (mean [SD], 3.5 [2.8] vs 4.7 [2.8]; difference, −1.23; 95% CI, −2.1 to −0.35; *P* = .006) compared with the usual care group. No significant differences were observed in other physician-reported outcomes. At 30-day follow-up, additional outcomes, including mean length of hospital stay, ICU admissions after discussion, readmissions, and 30-day postdischarge mortality, were similar between both groups (Table 2).

## Discussion

In this analysis of a cluster randomized trial, the use of a checklist that incorporates communication of poor prognosis for code status discussions among patients for whom CPR is considered futile led to a significant reduction in patient preferences for ICU admission and reduced residents’ difficulties in discussing the code status in these challenging conversations without measurable effects on patients’ psychosocial burden. These findings have important implications for advancing the quality and alignment of communication during code status discussions in this vulnerable patient population.

Discussing prognosis is central to effective code status discussions and essential for patient-centered advance care planning.^37^ However, in cases of probable CPR futility, often encountered in advanced chronic disease or palliative illness, these conversations become more complex. Unrealistic expectations regarding resuscitation outcomes are common among patients and families, and physicians may feel discomfort discussing end-of-life care or poor prognosis.^38,39^ The combination of misunderstanding and avoidance can delay or impair discussions, resulting in aggressive, misaligned end-of-life care, unnecessary interventions that prolong pain, and delayed palliative care integration.^40,41,42^

This study highlights the value of structured communication tools, such as checklists, in overcoming these barriers. Prior research^43^ showed that communication interventions can improve patients’ understanding of treatment options and outcomes in case of CPR, reducing CPR preferences, especially in patients with advanced chronic disease and severe illness. Grounded in shared decision-making principles, these approaches aim to align patient values with clinical recommendations.^44^

In patients for whom CPR is deemed futile, communication strategies must provide clear, realistic information about expected outcomes, including current health status and poor prognosis, and align with patients’ values, goals, and preferences.^45^ This requires balancing difficult news with empathy, while ensuring patients feel supported in making informed decisions that respect their dignity and priorities.

Recent pilot research on code status discussions in chronically ill patients introduced an informed assent approach. This strategy excludes CPR as the default in cases where CPR is considered futile while emphasizing patient engagement and respect for individual values.^46^ This approach fostered a more realistic understanding of the situation and was associated with a lower preference for CPR, as shown by reduced DNR preference in the intervention group. Similarly, our study’s checklist prioritized transparent communication of the patient’s poor prognosis and the limited effectiveness of CPR in advanced illness.

Although this intervention did not result in a statistically significant difference in DNR orders in our trial, likely due to limited power and high baseline DNR rates, it significantly reduced patients’ preference for ICU admission. This finding is noteworthy because ICU admission preferences may shift the focus toward aggressive care at the end of life, possibly conflicting with patients’ goals and values. Reducing ICU admissions in these situations might prioritize comfort-focused care, expedite palliative care referrals, and ultimately improve the quality of end-of-life care.^47,48^

Discussing resuscitation and poor prognosis in the context of medical futility can be challenging and may raise concerns about increasing patients’ anxiety or distress. However, our trial demonstrated no difference in patients’ psychological burden, aligning with prior research^46^ that poor prognosis communication does not necessarily heighten anxiety. These encouraging results suggest that structured, empathetic communication can support communication without harming patients’ mental well-being. This finding reinforces the importance of framing such discussions within a compassionate and patient-centered communication style.

A notable benefit of the checklist-guided approach was the significant reduction in physicians’ perceived difficulty during code status discussions. This is critical because prior studies^49,50^ have highlighted the challenges that physicians, particularly residents, face when initiating these conversations. Lack of training, discomfort with emotional responses, and limited experience with end-of-life care can hinder effective communication. The structured framework of the checklist in our study likely helped residents approach discussions with greater confidence and clarity.^49,50^ Importantly, the intervention was supported by training and feedback sessions, which might have further enhanced its acceptance and effectiveness.

This result underscores the need for incorporating structured communication training into medical education and residency programs, particularly in settings involving advance care planning and end-of-life care. Communication tools such as checklists, combined with training, may help standardize high-quality discussions and address the variability introduced by individual physicians’ communication styles, comfort levels, and experiences.

### Limitations

This study has several limitations, including the small sample size, along with a smaller than anticipated difference in DNR preferences between groups. This finding was largely due to high rates of DNR preferences in the control group, likely reflecting their substantial illness burden and resulting in a ceiling effect. In addition, a high proportion of patients were excluded due to cognitive or physical impairments, possibly causing selection bias. This issue highlights the vulnerability of this patient population and the need for implementation of early advanced care planning interventions and communication strategies for code status discussions involving the next of kin, ideally before patients lose decision-making capacity.^51^ Involving surrogate decision-makers early may mitigate some of these challenges, as suggested in critically ill patients.^52^ The small sample size limited the statistical power of our study and restricted the ability to perform multivariable modeling to account for confounders in this sample. As a result, our findings should be considered exploratory and primarily hypothesis-generating. Additionally, our study was conducted in Swiss teaching hospitals, which may limit its generalizability to other health care systems and cultural contexts. Variability in residents’ adherence to the checklist protocol and differences in their baseline communication skills may have further influenced the intervention’s effectiveness. Furthermore, because the GO-FAR score was developed more than a decade ago, improvements in CPR outcomes since then may affect its current calibration. Our use of a GO-FAR score threshold of 14 or higher should be interpreted cautiously. Clearly, larger multicenter trials are needed to confirm our findings and evaluate the intervention’s broader applicability. Future studies should also investigate whether similar approaches can improve outcomes for surrogate decision-makers and address other aspects of patient care, such as alignment with long-term goals of care and quality of life.

## Conclusions

In this analysis of a randomized clinical trial, the use of a checklist that incorporates communication of poor prognosis for code status discussions among patients for whom CPR is considered futile led to a significant reduction in patient preferences for ICU admission and eased this challenging code status discussions for residents. Importantly, the intervention did not increase patients’ psychosocial burden associated with these challenging discussions when the checklist was used compared to usual care discussions, demonstrating its feasibility and acceptability in this vulnerable population. These findings suggest that a structured communication, coupled with training, may have played a crucial role in improving the quality of code status discussion in the context of medical futility. Further research is needed to validate these findings, explore their applicability across diverse settings, and investigate their impact on long-term patient and family outcomes.

## Notes

Supplement 1. **eMethods.** Definition of CPR-futility according to the GO-FAR score and Clinical Frailty Scale **eMethods 2.** Development of a communication checklist **eMethods 3.** Definition of outcomes assessed during the trial **eTable 1.** Excluded patients **eTable 2.** Missing baseline data gathered through screening of patient record **eTable 3.** Missing outcome data gathered through screening of patient record **eTable 4.** Missing outcome data gathered through patient interview **eTable 5.** Missing outcome data gathered through resident interview

Supplement 2.
Trial Protocol

Supplement 3.
Data Sharing Statement

## References

[1] Einstein DJ, Einstein KL, Mathew P. Dying for advice: code status discussions between resident physicians and patients with advanced cancer–a national survey. J Palliat Med. 2015;18(6):535-541. doi:10.1089/jpm.2014.0373 25984641

[2] Bedulli M, Falvo I, Merlani P, Hurst S, Fadda M. Obstacles to patient inclusion in CPR/DNAR decisions and challenging conversations: a qualitative study with internal medicine physicians in Southern Switzerland. PLoS One. 2023;18(3):e0282270. doi:10.1371/journal.pone.0282270 36947569 PMC10032495

[3] Stapleton RD, Ehlenbach WJ, Deyo RA, Curtis JR. Long-term outcomes after in-hospital CPR in older adults with chronic illness. Chest. 2014;146(5):1214-1225. doi:10.1378/chest.13-2110 25086252 PMC4219338

[4] Perkins GD, Cooke MW. Variability in cardiac arrest survival: the NHS ambulance service quality indicators. Emerg Med J. 2012;29(1):3-5. doi:10.1136/emermed-2011-200758 22045608

[5] Meaney PA, Nadkarni VM, Kern KB, Indik JH, Halperin HR, Berg RA. Rhythms and outcomes of adult in-hospital cardiac arrest. Crit Care Med. 2010;38(1):101-108. doi:10.1097/CCM.0b013e3181b43282 19770741

[6] Girotra S, Nallamothu BK, Spertus JA, Li Y, Krumholz HM, Chan PS; American Heart Association Get with the Guidelines–Resuscitation Investigators. Trends in survival after in-hospital cardiac arrest. N Engl J Med. 2012;367(20):1912-1920. doi:10.1056/NEJMoa1109148 23150959 PMC3517894

[7] Amacher S, Sahmer C, Becker C, Post-intensive care syndrome and health-related quality of life in long-term survivors of cardiac arrest: a prospective cohort study. Sci Rep. 2024;14:s41598-024-61146-8. doi:10.1038/s41598-024-61146-8 PMC11079009 38719863

[8] Swiss Academies of Arts and Sciences. Wirkungslosigkeit und Aussichtslosigkeit – zum Umgang mit dem Konzept der Futility in der Medizin. Accessed December 5, 2025. https://www.samw.ch/en/Publications/Recommendations.html

[9] Truog RD. Is it always wrong to perform futile CPR? N Engl J Med. 2010;362(6):477-479. doi:10.1056/NEJMp0908464 20147712

[10] Ebell MH, Afonso AM. Pre-arrest predictors of failure to survive after in-hospital cardiopulmonary resuscitation: a meta-analysis. Fam Pract. 2011;28(5):505-515. doi:10.1093/fampra/cmr023 21596693

[11] de Vos R, de Haes HC, Koster RW, de Haan RJ. Quality of survival after cardiopulmonary resuscitation. Arch Intern Med. 1999;159(3):249-254. doi:10.1001/archinte.159.3.249 9989536

[12] Schneiderman LJ. Defining medical futility and improving medical care. J Bioeth Inq. 2011;8(2):123-131. doi:10.1007/s11673-011-9293-3 21765643 PMC3106156

[13] Schneiderman LJ, Jecker NS, Jonsen AR. Medical futility: its meaning and ethical implications. Ann Intern Med. 1990;112(12):949-954. doi:10.7326/0003-4819-112-12-949 2187394

[14] Lauridsen KG, Djärv T, Breckwoldt J, Tjissen JA, Couper K, Greif R; Education, Implementation and Team Task Force of the International Liaison Committee on Resuscitation (ILCOR). Pre-arrest prediction of survival following in-hospital cardiac arrest: a systematic review of diagnostic test accuracy studies. Resuscitation. 2022;179:141-151. doi:10.1016/j.resuscitation.2022.07.041 35933060

[15] Ebell MH, Jang W, Shen Y, Geocadin RG; Get With the Guidelines–Resuscitation Investigators. Development and validation of the Good Outcome Following Attempted Resuscitation (GO-FAR) score to predict neurologically intact survival after in-hospital cardiopulmonary resuscitation. JAMA Intern Med. 2013;173(20):1872-1878. doi:10.1001/jamainternmed.2013.10037 24018585

[16] Rockwood K, Song X, MacKnight C, . A global clinical measure of fitness and frailty in elderly people. CMAJ. 2005;173(5):489-495. doi:10.1503/cmaj.050051 16129869 PMC1188185

[17] Beck K, Vincent A, Cam H, . Medical futility regarding cardiopulmonary resuscitation in in-hospital cardiac arrests of adult patients: a systematic review and Meta-analysis. Resuscitation. 2022;172:181-193. doi:10.1016/j.resuscitation.2021.11.041 34896244

[18] Hu FY, Streiter S, O’Mara L, . Frailty and survival after in-hospital cardiopulmonary resuscitation. J Gen Intern Med. 2022;37(14):3554-3561. doi:10.1007/s11606-021-07199-1 34981346 PMC9585129

[19] Rubins JB, Kinzie SD, Rubins DM. Predicting outcomes of in-hospital cardiac arrest: retrospective US validation of the Good Outcome Following Attempted Resuscitation Score. J Gen Intern Med. 2019;34(11):2530-2535. doi:10.1007/s11606-019-05314-x 31512185 PMC6848295

[20] Ohlsson MA, Kennedy LM, Ebell MH, Juhlin T, Melander O. Validation of the good outcome following attempted resuscitation score on in-hospital cardiac arrest in southern Sweden. Int J Cardiol. 2016;221:294-297. doi:10.1016/j.ijcard.2016.06.146 27404694

[21] Piscator E, Göransson K, Bruchfeld S, . Predicting neurologically intact survival after in-hospital cardiac arrest-external validation of the Good Outcome Following Attempted Resuscitation score. Resuscitation. 2018;128:63-69. doi:10.1016/j.resuscitation.2018.04.035 29723607

[22] Thai TN, Ebell MH. Prospective validation of the Good Outcome Following Attempted Resuscitation (GO-FAR) score for in-hospital cardiac arrest prognosis. Resuscitation. 2019;140:2-8. doi:10.1016/j.resuscitation.2019.05.002 31078496

[23] Yamamoto R, Tamura T, Haiden A, ; SOS-KANTO 2017 Study Group. Frailty and neurologic outcomes of patients resuscitated from nontraumatic out-of-hospital cardiac arrest: a prospective observational study. Ann Emerg Med. 2023;82(1):84-93. doi:10.1016/j.annemergmed.2023.02.009 36964008

[24] Hamlyn J, Lowry C, Jackson TA, Welch C. Outcomes in adults living with frailty receiving cardiopulmonary resuscitation: a systematic review and meta-analysis. Resusc Plus. 2022;11:100266. doi:10.1016/j.resplu.2022.100266 35812717 PMC9256816

[25] Downar J, Hawryluck L. What should we say when discussing “code status” and life support with a patient? a Delphi analysis. J Palliat Med. 2010;13(2):185-195. doi:10.1089/jpm.2009.0269 19929226

[26] Kronick SL, Kurz MC, Lin S, . Part 4: systems of care and continuous quality improvement: 2015 American Heart Association guidelines update for cardiopulmonary resuscitation and emergency cardiovascular care. Circulation. 2015;132(18)(suppl 2):S397-S413. doi:10.1161/CIR.0000000000000258 26472992

[27] Becker C, Gross S, Beck K, A randomized trial of shared decision-making in code status discussions. N Engl J Med Evid. 2025;4(5):EVIDoa2400422. doi:10.1056/EVIDoa2400422 40261118

[28] Hopewell S, Chan AW, Collins GS, . CONSORT 2025 statement: updated guideline for reporting randomized trials. Nat Med. 2025;31(6):1776-1783. doi:10.1038/s41591-025-03635-5 40229553

[29] Swiss Academies of Arts and Sciences. Kommunikation im medizinischen Alltag: Ein Leitfaden für die Praxis Schweizerische Akademie der Medizinischen Wissenschaften. Accessed August 7, 2025. doi:10.5281/ZENODO.3576261

[30] Charlson ME, Pompei P, Ales KL, MacKenzie CR. A new method of classifying prognostic comorbidity in longitudinal studies: development and validation. J Chronic Dis. 1987;40(5):373-383. doi:10.1016/0021-9681(87)90171-8 3558716

[31] Smith GB, Prytherch DR, Meredith P, Schmidt PE, Featherstone PI. The ability of the National Early Warning Score (NEWS) to discriminate patients at risk of early cardiac arrest, unanticipated intensive care unit admission, and death. Resuscitation. 2013;84(4):465-470. doi:10.1016/j.resuscitation.2012.12.016 23295778

[32] Zigmond AS, Snaith RP. The Hospital Anxiety and Depression Scale. Acta Psychiatr Scand. 1983;67(6):361-370. doi:10.1111/j.1600-0447.1983.tb09716.x 6880820

[33] Laux L, Glanzmann P, Schaffner P, Spielberger C. Das State-Trait-Angstinventar. Theoretische Grundlagen und Handanweisung; 1981.

[34] Brennan C, Worrall-Davies A, McMillan D, Gilbody S, House A. The Hospital Anxiety and Depression Scale: a diagnostic meta-analysis of case-finding ability. J Psychosom Res. 2010;69(4):371-378. doi:10.1016/j.jpsychores.2010.04.006 20846538

[35] Knowles KA, Olatunji BO. Specificity of trait anxiety in anxiety and depression: meta-analysis of the State-Trait Anxiety Inventory. Clin Psychol Rev. 2020;82:101928. doi:10.1016/j.cpr.2020.101928 33091745 PMC7680410

[36] Karlsson J, Hammarström E, Fogelkvist M, Lundqvist LO. Psychometric characteristics of the Hospital Anxiety and Depression Scale in stroke survivors of working age before and after inpatient rehabilitation. PLoS One. 2024;19(8):e0306754. doi:10.1371/journal.pone.0306754 39186737 PMC11346913

[37] Lelorain S. Discussing prognosis with empathy to cancer patients. Curr Oncol Rep. 2021;23(4):42. doi:10.1007/s11912-021-01027-9 33718973

[38] Jackson VA, Emanuel L. Navigating and communicating about serious illness and end of life. N Engl J Med. 2024;390(1):63-69. doi:10.1056/NEJMcp2304436 38118003

[39] Gross S, Amacher SA, Rochowski A, . “Do-not-resuscitate” preferences of the general Swiss population: results from a national survey. Resusc Plus. 2023;14:100383. doi:10.1016/j.resplu.2023.100383 37056958 PMC10085778

[40] Committee on Approaching Death, Institute of Medicine. *Dying in America: Improving Quality and Honoring Individual Preferences Near the End of Life.* National Academies Press; 2015. 25927121

[41] Wright AA, Zhang B, Ray A, . Associations between end-of-life discussions, patient mental health, medical care near death, and caregiver bereavement adjustment. JAMA. 2008;300(14):1665-1673. doi:10.1001/jama.300.14.1665 18840840 PMC2853806

[42] Cardona-Morrell M, Kim J, Turner RM, Anstey M, Mitchell IA, Hillman K. Non-beneficial treatments in hospital at the end of life: a systematic review on extent of the problem. Int J Qual Health Care. 2016;28(4):456-469. doi:10.1093/intqhc/mzw060 27353273

[43] Becker C, Lecheler L, Hochstrasser S, . Association of communication interventions to discuss code status with patient decisions for do-not-resuscitate orders: a systematic review and meta-analysis. JAMA Netw Open. 2019;2(6):e195033. doi:10.1001/jamanetworkopen.2019.5033 31173119 PMC6563579

[44] Kriston L, Scholl I, Hölzel L, Simon D, Loh A, Härter M. The 9-item Shared Decision Making Questionnaire (SDM-Q-9): development and psychometric properties in a primary care sample. Patient Educ Couns. 2010;80(1):94-99. doi:10.1016/j.pec.2009.09.034 19879711

[45] Gilligan T, Coyle N, Frankel RM, . Patient-clinician communication: American Society of Clinical Oncology consensus guideline. J Clin Oncol. 2017;35(31):3618-3632. doi:10.1200/JCO.2017.75.2311 28892432

[46] Stapleton RD, Ford DW, Sterba KR, . Evolution of investigating informed assent discussions about CPR in seriously ill patients. J Pain Symptom Manage. 2022;63(6):e621-e632. doi:10.1016/j.jpainsymman.2022.03.009 35595375 PMC9179950

[47] Romano AM, Gade KE, Nielsen G, . Early palliative care reduces end-of-life intensive care unit (ICU) use but not ICU course in patients with advanced cancer. Oncologist. 2017;22(3):318-323. doi:10.1634/theoncologist.2016-0227 28220023 PMC5344633

[48] Lee RY, Brumback LC, Sathitratanacheewin S, . Association of physician orders for life-sustaining treatment with ICU admission among patients hospitalized near the end of life. JAMA. 2020;323(10):950-960. doi:10.1001/jama.2019.22523 32062674 PMC7042829

[49] Sulmasy DP, Sood JR, Ury WA. Physicians’ confidence in discussing do not resuscitate orders with patients and surrogates. J Med Ethics. 2008;34(2):96-101. doi:10.1136/jme.2006.019323 18234947

[50] Rosenberg LB, Greenwald J, Caponi B, . Confidence with and barriers to serious illness communication: a national survey of hospitalists. J Palliat Med. 2017;20(9):1013-1019. doi:10.1089/jpm.2016.0515 28375816

[51] Hui D, Kim SH, Roquemore J, Dev R, Chisholm G, Bruera E. Impact of timing and setting of palliative care referral on quality of end-of-life care in cancer patients. Cancer. 2014;120(11):1743-1749. doi:10.1002/cncr.28628 24967463 PMC4073257

[52] Bibas L, Peretz-Larochelle M, Adhikari NK, . Association of surrogate decision-making interventions for critically ill adults with patient, family, and resource use outcomes: a systematic review and meta-analysis. JAMA Netw Open. 2019;2(7):e197229. doi:10.1001/jamanetworkopen.2019.7229 31322688 PMC6646989

