# Supplementary material for: Checklist-Guided Code Status Discussions in Patients for Whom Cardiopulmonary Resuscitation Is Considered Futile: An Analysis of a Randomized Clinical Trial
Source: JAMA Netw Open. 2025 Sep 25;8(9):e2533638. doi: 10.1001/jamanetworkopen.2025.33638 (PMC12464790; doi:10.1001/jamanetworkopen.2025.33638)
Supplement: Supplement 1. — eMethods. Definition of CPR-futility according to the GO-FAR score and Clinical Frailty Scale eMethods 2. Development of a communication checklist eMethods 3. Definition of outcomes assessed during the trial eTable 1. Excluded patients eTable 2. Missing baseline data gathered through screening of patient record eTable 3. Missing outcome data gathered through screening of patient record eTable 4. Missing outcome data gathered through patient interview eTable 5. Missing outcome data gathered through resident interview [file jamanetwopen-e2533638-s001.pdf]

Supplemental Online Content

Arpagaus A, Arpagaus L, Becker C, et al. Checklist-guided code status discussions in patients for whom cardiopulmonary resuscitation is considered futile: an analysis of a randomized clinical trial. *JAMA Netw Open*. 2025;8(9):e2533638. doi:10.1001/jamanetworkopen.2025.33638

eMethods 1. Definition of CPR-futility according to the GO-FAR score and Clinical Frailty Scale

eMethods 2. Development of a communication checklist

eMethods 3. Definition of outcomes assessed during the trial

eTable 1. Excluded patients

eTable 2. Missing baseline data gathered through screening of patient record

eTable 3. Missing outcome data gathered through screening of patient record

eTable 4. Missing outcome data gathered through patient interview

eTable 5, Missing outcome data gathered through resident interview

This supplemental material has been provided by the authors to give readers additional information about their work.

eMethods 1: Definition of CPR-futility according to the GO-FAR score and Clinical Frailty Scale

CPR futility was defined as a GO-FAR score ≥ 14 (indicating a chance of survival with minimal neurological disability <1.7%) or patients in categories 7-9 in the Clinical Frailty Scale (indicating severe frailty with debilitating chronic conditions). To date, the GO-FAR score is the most extensively researched tool showing a good prognostic accuracy[1-4]. This clinical score predicts the probable chance of neurologically intact survival for patients following in-hospital resuscitation defined as a cerebral performance category (CPC) 1 by establishing cut-off points derived from a multivariate prediction model[5]. The lowest category indicates a very low (<1.7%) likelihood of survival with a favorable neurological outcome. Given that a survival chance of <1% is commonly adopted in medical research as a criterion for futility, it has been proposed that patients falling into this category should be regarded as CPR futile[6-8]. The CFS is a 9-category scale combining cognitive and physical dimensions of frailty, including comorbidity, cognitive decline, and disability. Studies have demonstrated that this scale, which is increasingly used in routine screening of older patients, is predictive for short-term mortality[9,10]. Furthermore, higher CFS scores are markedly associated with reduced survival in severe frailty (CFS 7-9)[11,12].

eMethods 2: Development of a communication checklist

Based upon a recently published communication checklist designed for shared decision-making in code status discussions and insights gained from clinical encounters with patients and physicians, we have developed a tailored checklist specifically for discussion of code status in patients in whom CPR is considered futile.[13]

This structured communication approach aims to guide and support healthcare providers in navigating sensitive conversations about resuscitation decisions, particularly when CPR is deemed medically inappropriate based on clinical and prognostic evaluations. The checklist emphasizes empathy, clear communication of the prognosis both in general and in the context of the patient's specific medical condition, and thorough documentation of the discussion.

The checklist is organized into five key sections: '**G**ive routine information', '**U**nderline situation', '**I**nterest in patients' thoughts and views', '**D**ocument decision', and '**E**xplore advances directives'. Each section is designed to balance high information content and the acknowledgment of patients' emotions. The initials of the five sections create the acronym '**GUIDE** Decision', highlighting the importance of guiding the physicians through this complex discussion with their patients.

The '**G**ive routine information' section ensures that patients understand these discussions and are conducted routinely with all patients upon hospital admission as a standard part of clinical care. It clarifies that these conversations have no impact on ongoing treatment except in the context of resuscitation attempts.

In the '**U**nderline situation' section, the general poor prognosis associated with cardiac arrest with a low rate of survivors and potential for significant neurological impairments following CPR is highlighted. For patients with advanced illnesses, clinicians explain the high likelihood of unsuccessful resuscitation and the possibility of prolonged suffering without improved survival odds. This discussion leads to the recommendation not to pursue resuscitation measures and instead focus on palliative care.

The '**I**nterest in patients' thoughts and views' section addresses patients' reaction to the recommendation against resuscitation measures. Using patient-centered communication techniques, such as the "NURSE" techniques (referring to Naming,

Understanding, Respecting, Supporting, Exploring) clinicians acknowledge emotions, explore the patient's expectations, health beliefs, and lifestyle priorities, and assess their understanding of the situation. This empathetic approach fosters trust and ensures the patient feels heard and supported.

The '**D**ocument decision' section provides specific recommendations regarding intubation and intensive care based on the patient's medical condition. To ensure that the patients' code status is clearly communicated, and their wishes are respected in the event of cardiac arrest, the decision is documented in the electronic medical record accordingly.

Finally, in the '**E**xplore advances directives' clinicians review whether the patient has previously established an advance directive. If an advance directive exists, its content is evaluated to ensure alignment with the patients' current wishes. In cases of discrepancy, clinicians recommend updating the directive to reflect the patient's preferences accurately.

eMethods 3: Definition of outcomes assessed during the trial

The **primary endpoint** was defined as frequency of DNR Code Status compared to CPR Code Status among patients per resident (i.e., patients choosing that CPR, mechanical ventilation and intensive care measures should not be performed in case of acute deterioration).

The **secondary endpoints** were defined as follows:

1. **Patient-associated outcomes**
    a. Patients' concerns and fears raised during the resuscitation discussion: Specifically, we assessed the following dimensions, each rated on a visual analog scale (VAS) from 0 to 10: (1) the level of disturbance

caused by the discussion, (2) fear of suffering from an actual cardiac arrest, (3) fear of suffering from a life-threatening disease (4) perceived pressure felt during discussion.

b. Patients' psychological burden assessed by the validated German translation of the Hospital Anxiety and Depression Scale (HADS).[14] The HADS is a widely used screening tool designed to detect anxiety and depression in hospital settings. It consists of 14 items, divided into two subscales: 7 items for anxiety (HADS-A) and 7 items for depression (HADS-D). Each item is scored on a 4-point scale (0-3), with total scores for each subscale ranging from 0 to 21. Scores of 0-7 indicate normal levels, 8-10 suggest borderline cases, and 11-21 indicate clinical levels of anxiety or depression. The HADS score is valued for its simplicity, reliability, and sensitivity in identifying emotional disorders in medically ill patients.

c. **Imputation of missing data of the HADS-Score**

   Missing data in the HADS-Score were handled according to the guidelines of the official German translation manual[14]. If more than one item was missing in a subscale (HADS-D, HADS-A), the entire subscale was excluded from analysis. If only one item was missing, its value was imputed by calculating the mean score of the remaining items on that subscale.

d. **Patients' concerns and fears**

   Patients' concerns and fears were assessed using the validated German translation of the State-Trait Anxiety Inventory (STAI). The State-Trait Anxiety Inventory (STAI) is a psychological inventory that measures

anxiety in adults.[15] It differentiates between two types of anxiety: state anxiety (temporary, situational anxiety) and trait anxiety (general, long-term anxiety). The inventory consists of 40 self-report items, split evenly into two 20-item subscales: 20 for state anxiety (S-Anxiety) and 20 for trait anxiety (T-Anxiety). Participants rate their feelings on a 4-point Likert scale with subscale ranging from 20 to 80. Higher scores indicate greater anxiety. The STAI is widely regarded for its reliability and ability to differentiate between transient and chronic anxiety states in clinical and research settings.

e. **Imputation of missing data of the STAI-Score**

Missing data in the STAI scores were managed following the official manual.[15] Respondents who omitted more than two items on either scale (S-Anxiety, T-Anxiety) were excluded. For those with fewer missing items, a prorated full-scale score was calculated. The mean weighted score of the completed items was multiplied by 20 and rounded to the nearest whole number.

2. Physician-associated outcomes

a. Physicians' perception of the resuscitation discussion: physicians` perceptions were assessed separately for both study groups regarding the following dimensions (each rated on a VAS (0-10)): (1) Satisfaction with resuscitation discussion and self-confidence during the discussion. (2) Perceived time management of the discussion. (3) Physicians' difficulty of the discussion.

3. Documented results from database at 30-day follow-up

a. Length of patient's hospital stay (in days)
b. Whether the patient was admitted to the intensive care unit admission after the resuscitation discussion
c. Whether the patient was readmitted to hospital within 30 days
d. Whether the patient died within 30 days

eTable 1: Excluded patients

Overall, 62.5% (295/472) of screened CPR-futile patients were excluded. Of these, 58.1% (274/472) met the exclusion criteria, and 4.4% (21/472) were not available during the study period or did not speak local language. Most excluded patients, 49.6% (234/472), were excluded due to cognitive impairment. Further assessments were feasible in 43.5% (77/177) patients, while 20% (37/177) declined an interview, 14.6% (26/177) had cognitive or physical impairment and 8% (14/177) were unavailable. A detailed overview of the excluded patients and missing data is provided in following **eTables 1-5**.

	Usual Care Group	Checklist group
**N, No. (%)**	**119**	**155**
**Exclusion criteria**		
**Intoxication**	2 (1.6%)	4 (2.5%)
**Paracusis**	2 (1.6%)	3 (1.9%)
**Serious psychiatric conditions**	5 (4.2%)	11 (7.0%)
**Cognitive impairment/Clinical deterioration with cognitive impairment**	102 (85.7%)	132 (85.1%)
**Already included in study**	6 (5.0%)	4 (2.5%)
**Organizational issues**	2 (1.6%)	1 (1.0%)

Legend: Patients excluded according to exclusion criteria n = 274. Patients excluded for secondary assessment: Patients not available during study period n =14. Patients excluded not speaking a local language = 7.

eTable 2: Missing baseline data gathered through screening of patient record

Baseline Data - Patients				
**Variable**	**n total**	**n completed**	**n missings**	**Reasons for missing data**
**Age**	177	177	0	n.a.
**Gender**	177	177	0	n.a.

**Civil status**	177	174	3	not mentioned in electronic record
**Having children?**	177	174	3	not mentioned in electronic record
**Citizenship**	177	177	0	n.a.
**Religious affiliation**	177	175	2	not mentioned in electronic record
**Main diagnoses leading to hospitalization**	177	177	0	n.a.
**Comorbidities**	177	177	0	n.a.
**Charlson comorbidity index**	177	177	0	n.a.
**NEWS2-Score**	177	177	0	n.a.
**GOFAR Score**	177	177	0	n.a.
**Survival according to GOFAR Score**	177	177	0	n.a.
**Clinical Frailty Scale**	177	177	0	n.a.
**Baseline Data - Residents**				
**Variable**	**n total**	**n completed**	**n missings**	**Reasons for missing data**
**Age**	206	206	0	n.a.
**Sex**	206	206	0	n.a.
**Primary language**	206	206	0	n.a.
**Job Experience**	206	206	0	n.a.

Legend: n, number; n.a., not applicable; NEWS-2, National Early Warning Score-2; GOFAR, Good Outcome Following Attempted Resuscitation

eTable 3: Missing outcome data gathered through screening of patient record

Variable	n total	n completed	n missings	reasons for missing data
**Primary Endpoint**				
**Code Status preference**	177	177	0	n.a.
**Mechanical ventillation preference**	177	154	23	not documented in patient record
**Intensive care unit preference**	177	170	7	not documented in patient record
**30d Assessment**				
**Duration of hospitalisation**	177	177	0	n.a.
**ICU stay (yes)**	177	177	0	no missings
**Readmission**	177	177	0	n.a.
**30d mortality**	177	177	0	n.a.

Legend: n, number; ICU, intensive care unit

eTable 4: Missing outcome data gathered through patient interview

Variable, No. (%)	n total	n interview	n completed	n missings	reasons for missing data	n patients
**Code Status preference**	177	100	91	9	patient did not want to respond / discontinues interview	2
					cognitive difficulties to complete interview	n.a.
					physical difficulties to complete interview (e.g., tracheostomy, fatigue, terminally ill, dead before interview)	2
					language difficulties to complete interview	1
					patient does not remember code status discussion or information	n.a.
					other/unclear	4
**Mechanical ventillation preference**	177	100	87	13	patient did not want to respond / discontinues interview	3
					cognitive difficulties to complete interview	n.a.
					physical difficulties to complete interview (e.g., tracheostomy, fatigue, terminally ill, dead before interview)	2
					language difficulties to complete interview	2
					patient does not remember code status discussion or information	n.a.
					other/unclear	6
**Intensive care unit preference**	177	100	90	10	patient did not want to respond / discontinues interview	2
					cognitive difficulties to complete interview	1
					physical difficulties to complete interview (e.g., tracheostomy, fatigue, terminally ill, dead before interview)	1
					language difficulties to complete interview	2

					patient does not remember code status discussion or information	n.a.
					other/unclear	4
						10
**Patients' disturbance caused by the discussion (VAS 0-10), mean (SD)**	177	100	81	19	patient did not want to respond / discontinues interview	1
					cognitive difficulties to complete interview	1
					physical difficulties to complete interview (e.g., tracheostomy, fatigue, terminally ill, dead before interview)	7
					language difficulties to complete interview	3
					patient does not remember code status discussion or information	n.a.
					other/unclear	7
**Patients' fear of suffering from an actual cardiac arrest (VAS 0-10), mean (SD)**	177	100	80	20	patient did not want to respond / discontinues interview	1
					cognitive difficulties to complete interview	1
					physical difficulties to complete interview (e.g., tracheostomy, fatigue, terminally ill, dead before interview)	6
					language difficulties to complete interview	3
					patient does not remember code status discussion or information	n.a.
					other/unclear	9
**Patients' fear of suffering from a life-threatening disease (VAS 0-10), mean (SD)**	177	100	80	20	patient did not want to respond / discontinues interview	1

					cognitive difficulties to complete interview	1
					physical difficulties to complete interview (e.g., tracheostomy, fatigue, terminally ill, dead before interview)	6
					language difficulties to complete interview	3
					patient does not remember code status discussion or information	n.a.
					other/unclear	9
**Patients' perceived feeling of being put under pressure during discussion (VAS 0-10), mean (SD)**	177	100	79	21	patient did not want to respond / discontinues interview	1
					cognitive difficulties to complete interview	1
					physical difficulties to complete interview (e.g., tracheostomy, fatigue, terminally ill, dead before interview)	6
					language difficulties to complete interview	4
					patient does not remember code status discussion or information	n.a.
					other/unclear	9
**State trait anxiety and depression inventory, mean, SD**	177	100	77	23	patient did not want to respond / discontinues interview	2
					cognitive difficulties to complete interview	1
					physical difficulties to complete interview (e.g., tracheostomy, fatigue, terminally ill, dead before interview)	6
					language difficulties to complete interview	3
					patient does not remember code status discussion or information	1
					other/unclear	10

**Hospital anxiety and depression scale - Anxiety over cut-off 8, yes**	177	100	75	25	patient did not want to respond / discontinues interview	3
					cognitive difficulties to complete interview	1
					physical difficulties to complete interview (e.g., tracheostomy, fatigue, terminally ill, dead before interview)	8
					language difficulties to complete interview	3
					patient does not remember code status discussion or information	n.a.
					other/unclear	10
**Hospital anxiety and depression scale- Depression over cut-off 8, yes**	177	100	73	27	patient did not want to respond / discontinues interview	3
					cognitive difficulties to complete interview	2
					physical difficulties to complete interview (e.g., tracheostomy, fatigue, terminally ill, dead before interview)	7
					language difficulties to complete interview	3
					patient does not remember code status discussion or information	2
					other/unclear	10
**Hospital anxiety and depression scale total, mean, (SD)**	177	100	73	27	patient did not want to respond / discontinues interview	2
					cognitive difficulties to complete interview	2
					physical difficulties to complete interview (e.g., tracheostomy, fatigue, terminally ill, dead before interview)	7
					language difficulties to complete interview	3

					patient does not remember code status discussion or information	2
					other/unclear	11
**EQindex, mean (SD)**	177	100	91	9	patient did not want to respond / discontinues interview	1
					cognitive difficulties to complete interview	n.a.
					physical difficulties to complete interview (e.g., tracheostomy, fatigue, terminally ill, dead before interview)	1
					language difficulties to complete interview	1
					patient does not remember code status discussion or information	n.a.
					other/unclear	6
**EQvas, mean (SD)**	177	100	87	13	patient did not want to respond / discontinues interview	1
					cognitive difficulties to complete interview	n.a.
					physical difficulties to complete interview (e.g., tracheostomy, fatigue, terminally ill, dead before interview)	4
					language difficulties to complete interview	2
					patient does not remember code status discussion or information	n.a.
					other/unclear	6

Legend: n, number; VAS, visual analogue scale

eTable 5: Missing outcome data gathered through resident interview

Variable	n total	n completed	n missings	reasons for missing data	n patients
**Physicians' overall satisfaction with resuscitation discussion (VAS 0-10), mean (SD)**	177	159	18	other/no reason given	17
				resident did not remember the code status discussion	1
**Physicians' perceived time management of the discussion (VAS 0-10), mean (SD)**	177	159	18	other/no reason given	17
				resident did not remember the code status discussion	1
**Physicians' perceived difficulty of the discussion (VAS 0-10), mean (SD)**	177	159	18	other/no reason given	17
				resident did not remember the code status discussion	1

Legend: n, number; VAS, visual analogue scale

References

1. Amacher SA, Blatter R, Briel M, et al. Predicting neurological outcome in adult patients with cardiac arrest: systematic review and meta-analysis of prediction model performance. *Crit Care*. Dec 11 2022;26(1):382. doi:10.1186/s13054-022-04263-y
2. Piscator E, Göransson K, Forsberg S, et al. Prearrest prediction of favourable neurological survival following in-hospital cardiac arrest: The Prediction of outcome for In-Hospital Cardiac Arrest (PIHCA) score. *Resuscitation*. Oct 2019;143:92-99. doi:10.1016/j.resuscitation.2019.08.010
3. Rubins JB, Kinzie SD, Rubins DM. Predicting Outcomes of In-Hospital Cardiac Arrest: Retrospective US Validation of the Good Outcome Following Attempted Resuscitation Score. *J Gen Intern Med*. Nov 2019;34(11):2530-2535. doi:10.1007/s11606-019-05314-x
4. Grandbois van Ravenhorst C, Schluep M, Endeman H, Stolker R-J, Hoeks SE. Prognostic models for outcome prediction following in-hospital cardiac arrest using pre-arrest factors: a systematic review, meta-analysis and critical appraisal. *Critical Care*. 2023/01/20 2023;27(1):32. doi:10.1186/s13054-023-04306-y
5. Ebell MH, Jang W, Shen Y, Geocadin RG. Development and validation of the Good Outcome Following Attempted Resuscitation (GO-FAR) score to predict neurologically intact survival after in-hospital cardiopulmonary resuscitation. *JAMA Intern Med*. Nov 11 2013;173(20):1872-8. doi:10.1001/jamainternmed.2013.10037
6. Schneiderman LJ, Jecker NS, Jonsen AR. Medical futility: its meaning and ethical implications. *Ann Intern Med*. Jun 15 1990;112(12):949-54. doi:10.7326/0003-4819-112-12-949
7. Mancini ME, Diekema DS, Hoadley TA, et al. Part 3: Ethical Issues: 2015 American Heart Association Guidelines Update for Cardiopulmonary Resuscitation and Emergency Cardiovascular Care. *Circulation*. Nov 3 2015;132(18 Suppl 2):S383-96. doi:10.1161/cir.0000000000000254
8. Morrison LJ, Kierzek G, Diekema DS, et al. Part 3: Ethics. *Circulation*. 2010;122(18_suppl_3):S665-S675. doi:doi:10.1161/CIRCULATIONAHA.110.970905
9. Gregorevic KJ, Hubbard RE, Lim WK, Katz B. The clinical frailty scale predicts functional decline and mortality when used by junior medical staff: a prospective cohort study. *BMC Geriatr*. Jun 2 2016;16:117. doi:10.1186/s12877-016-0292-4
10. Chua XY, Toh S, Wei K, Teo N, Tang T, Wee SL. Evaluation of clinical frailty screening in geriatric acute care. *J Eval Clin Pract*. Feb 2020;26(1):35-41. doi:10.1111/jep.13096
11. Hamlyn J, Lowry C, Jackson TA, Welch C. Outcomes in adults living with frailty receiving cardiopulmonary resuscitation: A systematic review and meta-analysis. *Resusc Plus*. Sep 2022;11:100266. doi:10.1016/j.resplu.2022.100266
12. Ibitoye SE, Rawlinson S, Cavanagh A, Phillips V, Shipway DJH. Frailty status predicts futility of cardiopulmonary resuscitation in older adults. *Age and Ageing*. 2020;50(1):147-152. doi:10.1093/ageing/afaa104
13. Becker C, Gross S, Beck K, et al. A Randomized Trial of Shared Decision-Making in Code Status Discussions. *NEJM Evid*. May 2025;4(5):EVIDoa2400422. doi:10.1056/EVIDoa2400422
14. Christoph Herrmann-Lingen UB, R. Philip Snaith. Hospital Anxiety and Depression Scale - Deutsche Version 2018;(4., aktualisierte und neu normierte Auflage 2018)
15. Spielberger C, Gorsuch R, Lushene R, Vagg PR, Jacobs G. *Manual for the State-Trait Anxiety Inventory (Form Y1 – Y2)*. vol IV. 1983.
