# Supplementary material for: Checklist-Guided Code Status Discussions in Patients for Whom Cardiopulmonary Resuscitation Is Considered Futile: An Analysis of a Randomized Clinical Trial
Source: JAMA Netw Open. 2025 Sep 25;8(9):e2533638. doi: 10.1001/jamanetworkopen.2025.33638 (PMC12464790; doi:10.1001/jamanetworkopen.2025.33638)
Supplement: Supplement 2. — Trial Protocol [file jamanetwopen-e2533638-s002.pdf]

Cluster-Randomized Trial of Checklist-guided Shared Decision-Making for Code Status Discussions in Medical Inpatients

The **CLEAR Checklist Trial** (**C**linician-Patient Engagement, **L**earn and Inform, **E**xplore Patient Preferences, **A**ssess and Document, **R**eview Advance Directives)

Study protocol and statistical analysis plan

Steering Committee:

Prof. Dr. med. Sabina Hunziker (Principal Investigator)
Medical Communication,
Department of Psychosomatic Medicine,
University Hospital Basel
CH - 4031 Basel
Switzerland
Email: Sabina.Hunziker@usb.ch

Dr. med. Christoph Becker
Department of Psychosomatic Medicine
Medical Communication,
University Hospital Basel
CH - 4031 Basel
Switzerland
Email: Christoph.Becker@usb.ch

Prof. Dr. med. Stefano Bassetti
Division of Internal Medicine,
University Hospital Basel
CH - 4031 Basel
Switzerland
Email: Stefano.Bassetti@usb.ch

Prof. Dr. med. Philipp Schütz
Division of Internal Medicine
Cantonal Hospital Aarau,
CH – 5001 Aarau,
Switzerland
Email: Philipp.schuetz@ksa.ch

Prof. Dr. med. Drahomir Aujesky,
Department of General Internal Medicine,
Inselspital Bern University Hospital,
CH-3010 Bern
Switzerland
Email: drahomir.aujesky@insel.ch

Clinical Trial Unit (CTU)
University Hospital Basel,
Spitalstrasse 12
CH - 4031 Basel
Switzerland

Table of contents

1. Summary of the research plan 3

2. Research plan 5

2.1. Current state of research in the field 5

2.2. Detailed research plan 7

2.2.1. Overall hypothesis and general aims 7

2.2.2. Setting and collaboration 7

2.2.3. Patient population, inclusion, and exclusion criteria 7

2.2.4. Informed consent statement 8

2.2.5. Study flow 8

2.2.6. Interventions 10

2.2.7. Patient involvement 11

2.2.8. Primary and secondary endpoints 11

2.3.9 Statistical approach 12

2.3.10 Power and sample size considerations 13

2.3.11 Recruitment and recruitment feasibility 13

2.3.12 Potential limitations 14

2.4 Relevance and impact 17

3 Bibliography 18

1. Summary of the research plan

Background: A patients' decision regarding "Do Not Resuscitate" (DNR) code status, which is a legal order to withhold cardiopulmonary resuscitation (CPR) or advanced cardiac life support in respect of a patient's wish in case of a cardiac arrest or respiratory failure, has important medical and socioeconomic consequences. Yet, hospitalized patients often have insufficient information about expected outcomes from resuscitation measures. Research shows that <20% of in-hospital patients, who require CPR survive and subsequently many have permanent brain damage or disability. Although challenging in clinical routine, sharing information about expected prognosis with patients is a prerequisite for informed decision-making. In a recent hospital-based survey we found that DNR discussions are often insufficient with physicians not actively involving patients in the decision-making process, but relying decisions on the presumed preferences without in-depth discussions of patients‘ choices and consequences.

General aim: This trial is designed to investigate whether checklist-guided shared decision-making including decision aids and communication of expected outcome influences patients' decision regarding DNR code status, and at the same time, improves decision-making quality as judged by patient's decisional comfort, patient knowledge and involvement in decision-making and patient satisfaction.

Patient population: Consecutive adult medical patients admitted for in-hospital care, independent of medical diagnosis are eligible. Patients unable to complete questionnaires or unable to follow code status discussions (e.g., due to cognitive impairment such as dementia or delirium) and patients in whom resuscitation efforts are deemed futile are excluded.

Methods: multicenter, cluster randomized controlled trial involving six Swiss teaching hospitals.

Intervention: Medical residents conducting code status discussions will be randomized by an electronic, web-based system to the intervention group with shared decision-making facilitated by a checklist and a decision aid or to the usual care group. The decision aid was developed during a consensus conference, in which feedback from patients and clinicians was sought and integrated, and later field-tested until thematic saturation was achieved. Intervention group residents will receive detailed instructions about the shared decision-making checklist with specific communication teachings. To reduce bias, control group residents will also receive information about the importance of code status discussions and a general communication training, but no teaching regarding shared decision-making and no checklist will be used.

Endpoints: The primary outcome is the frequency of DNR code status among patients per resident (i.e., patients choosing that CPR and intubation measures should not be performed in case of acute deterioration). Our key secondary endpoint is the quality of decision-making as judged by patient's decisional comfort assessed through the validated German translation of the Decision Conflict Scale. Additionally, we will investigate patient`s knowledge about resuscitation measures and expected outcome, patients' involvement in the shared decision-making process assessed through the validated German translation of the SDM-q-9 questionnaire, patients' concerns and fears and overall satisfaction with the code status discussion, as well as physicians perceived comfort with patient's choice and satisfaction with code status discussion. At hospital discharge, we will assess length of hospital stay, ICU-admissions, in-hospital resuscitations, through medical chart review.

Sample size and statistics: Based on pilot data in this patient population, we expect that 30% of patients in the usual care control will have a DNR code status, which will increase to 45% in the intervention group. We plan to include 174 residents (cluster) with a mean of 5 eligible patients per week over a 3-week period (total of 15 patients per cluster) in the five participating centers over a total study time of 2 years. Based on these assumptions, we aim to include 2610 patients among 174 residents, which will give this study an 80% power at a 0.05 alpha error with an inter-cluster correlation of 0.5.

Discussion: Although code status discussions are a cornerstone of patient-centered care, there is a lack of trials investigating the most appropriate approach for physicians to communicate with patients about their preference. This multicenter randomized-controlled trial will close this important knowledge gap by

systematically investigating effects of checklist-guided shared decision-making on patients' decisions regarding DNR status and the quality of decision-making.

2. Research plan

2.1. Current state of research in the field

Patient-centered care has become an important ethical principle in clinical practice over the last decades. The American Institute of Medicine considers patient-centered care as a key element of high-quality care[1]. Patients are seen as partners with healthcare professionals and their individual needs and health outcomes are the main focus in patient-centered care. Not only clinical markers but also psychological, emotional needs are of major importance in this context. Herein, a cornerstone of patient-centered care is the conversation with a patient about the preference of his code status. Informing patients about their choices of treatment, especially in elderly polymorbid patients, is acknowledged as an essential component of high-quality medical care[2-4]. Specifically, a patient's decision regarding "Do Not Resuscitate" (DNR) code status, which is a legal order to withhold cardiopulmonary resuscitation (CPR) or advanced cardiac life support (ACLS) in respect of the wishes of a patient in case their heart was to stop, or they were to stop breathing, has important medical and socioeconomic consequences. Research suggests that patients favor to be involved in medical decisions regarding their care at the end of life[5-8]. Still, although recommended by the Swiss Academy for Medical Sciences, such DNR discussions are often insufficient in clinical routine. In fact, in a recent survey focusing on code status discussions in Swiss hospitals we found that more than 60% of the surveyed patients reported that they do not recall such discussions, reflecting a deficiency in the quality of communication (Manuscript submitted for publication). In addition, research found that physicians often omit to describe resuscitation measures like chest compression or mechanical ventilation[9]. Although CPR is an invasive procedure with potential complications and research showing that less than 20% of in-hospital patients, who require CPR will survive and many have permanent brain damage or disability, risks and benefits are often not communicated with patients[10, 11], leading to misconceptions about CPR and expected outcome[12, 13]. Especially patients with low health literacy, who have difficulties to understand medical coherences, need improvements in communication to empower well-informed decision-making regarding code status.

There are several challenges to the current practice of code status discussions. First, these discussions are complex as objective medical facts and personal perspectives of physicians and patients both need to be integrated in the communication. Second, several studies found code status discussions to be ineffective due to poor communication skills of physicians[14, 15], which is particularly true for inexperienced residents[16]. Code status discussions are often conducted in a non-personalized way, missing the chance to focus on patients' values and goals[17]. Insufficient time during patient encounters pose an additional barrier for optimal communication[18-20]. Furthermore, the decision-making during code status discussions is often challenged by uncertainty around interventions and therapies that might be available, but whose outcomes remain uncertain[21, 22].

Importantly, patients with in-hospital cardiac arrests have a poor prognosis with a survival to hospital discharge rate of less than 20%[23, 24]. Furthermore, 40% of survivors suffer from substantial neurologic

deficits limiting their possibility to live an independent life[25]. In 2012, a British multi-center cohort study investigated medical charts of patients that had undergone resuscitation of an in-hospital cardiac arrest[26]. In more than 75% of patients that received CPR, the code status was unknown and 67% of patients that were resuscitated, actually had an underlying pre-existing fatal disease. Importantly, an independent assessment of all cases found that a DNR status would have been appropriate in 85% of cases, because the risk / benefit ratio was unfavourable for the patient, indicating medical futility. In addition, a recent cancer study shows that physicians often rather document a presumed code status, than conduct a discussion, leading to a high proportion of full code status despite severe illnesses of a patient [27]. Almost a third of patients that were presumably documented as full code would have preferred DNR code status[27].

Some previous studies aimed to improve code status discussions by investigating different communication elements. However, most studies that investigated different approaches to improve communication were observational and some relied on physicians rating of their own performance[28-31]. Other studies focused on patients with life-sustaining therapy in ICU settings, with a focus on end-of-life decisions like withdrawal of therapy [32, 33]. Thus, little is known about how to best communicate with polymorbid patients, where death is not imminent. Moreover, interventions were often complex, limiting clinical practicability. Best results regarding patients' knowledge of resuscitation were found in studies that used short videos about CPR as decision aids[34-37]. Although videos seem to increase patients' knowledge effectively and may therefore facilitate end-of life discussions, their practicality in elderly, polymorbid patients is unclear and they do not allow for personalized decisions. Importantly, most studies that investigated different approaches to facilitate code status discussions focused on terminally ill patients, suffering from advanced cancer stages or other palliative diseases. Still, due to its unpredictable nature, code status discussions are important for all patients admitted to hospital as cardiopulmonary arrest occurs in almost 1 per 1000 hospitalization days[38]. Above all, despite all research efforts, physicians' common practice to determine a presumed code status without prior discussion has not changed over the last 20 years, when an American trial, which investigated different approaches to improve care for seriously ill patients, pointed out this deficiency of medical practice[39].

In recent years, shared decision-making has become the standard for communication in situations of equal treatment options (equipoise). The goal of shared decision-making is to inform patients with best possible evidence and consider their perceptions and preferences. Through this process, the informed patient is empowered to weigh the different treatment options and influence medical decisions actively. Studies found that shared decision-making leads to higher satisfaction rates and increases self-responsibility of patients[40]. A recent randomized trial compared the effectiveness of shared decision-making with usual care in choice of admission for observation and further cardiac testing or for referral to outpatient evaluation in patients with chest pain and possible ischemic heart disease[40]. The trial found that shared decision-making, facilitated by the use of a decision aid, increased patient knowledge about their risk, increased engagement, reduced decisional conflict and safely decreased the rate of admission to an observation unit for further cardiac testing. Importantly, there is a relevant lack of trials looking at the effects of shared

decision-making in the setting of code status discussions (with patients in whom resuscitation is an option, i.e. who are not medically futile). Herein, we hypothesize that checklist-guided shared decision-making compared to routine will increase the frequency of patients choosing DNR code status. We also hypothesize that the intervention will not increase fear or dissatisfaction, but will lead to a higher comfort with the code status choice, improve satisfaction of patients across different quality dimensions and knowledge regarding resuscitation

2.2. Detailed research plan

2.2.1. Overall hypothesis and general aims

The overall aim of this multicenter, cluster randomized-controlled trial is to investigate whether checklist-guided shared decision-making including decision aids and communication of expected outcome influences patients` decision regarding DNR code status, and at the same time improves decision-making quality as judged by patient decisional comfort, patient knowledge, and involvement in decision-making and patient satisfaction. As recommended for interventional trials, the study protocol was published at clinicaltrials.gov (https://clinicaltrials.gov/ct2/show/NCT03872154).

2.2.2. Setting and collaboration

This multicenter study will be conducted in six participating Swiss teaching hospitals, namely the University Hospital Basel, the Kantonsspital Aarau, the University Hospital Bern (Inselspital), the Cantonal Hospital Baselland Liestal, the St.Claraspital Basel and the Cantonal Hospital Lucerne. The head of departments at the different locations have agreed to give full support for this study. The local investigators will support the study with personal resources and by including patients. Our main study team from the University Hospital Basel will be responsible for the study set-up, recruitment and training of the study nurses, who support data collection and data cleaning as part of the ancillary project.

2.2.3. Patient population, inclusion, and exclusion criteria

Residents on the medical wards will be the primary level of randomization. We have no exclusion criteria for physicians except if they refuse participation. Outcomes will be assessed in patients treated by these physicians. Any adult (>18 years) patient that is admitted for in-hospital care will be eligible. There will be no exclusion based on the type of illness, comorbidity or severity of illness in order to include a representative population of patients in Swiss hospitals. We will exclude patients unable to complete questionnaires or unable to follow code status discussions due to (1) intoxication, (2) paracusis; (3) serious psychiatric conditions (e.g., psychosis, depression with suicidal tendency, stupor), (4) cognitive impairment (e.g. dementia, delirium). Also, patients prior included in this study (i.e., patients who are hospitalized for the second time) are not eligible. Patients, in whom resuscitation attempts would be futile, will not be included in this study.

Recently Ebell et al developed the GO-FAR score, a clinical risk score to predict the estimated likelihood of neurologically intact survival of patients after in-hospital resuscitation[41]. The authors developed a multivariate prediction model based on 51`240 patients who experienced in-hospital cardiac arrest and calculated cut-off scores indicating likelihood of functional survival. The two lowest categories represent very low (<1%) and low (1-3%) likelihood of survival. It has therefore been suggested that these patients should be considered futile regarding CPR attempts [42]. Another tool to assess whether patients are at increased risk for poor outcome is the Clinical Frailty Scale[43]. This 9-category scale combines cognitive and physical aspects of frailty such as comorbidity, cognitive impairment, and disability. Research has shown that the scale, which is increasingly used in routine screening of older patients, predicts short-term mortality[44, 45]. However, the prevalence of futility in hospitalized patients upon admission assessed through the GO-FAR score and the Clinical Frailty Scale has not been systematically investigated.

According to the literature, we will define futility as a GO-FAR score ≥ 14 or patients in categories 7-9 in the Clinical frailty scale.

2.2.4. Informed consent statement

The study was approved by the local Ethics Committee (Ethics Committee northwest/central Switzerland; Ethikkommission Nordwest- und Zentralschweiz; EKNZ as well as the Ethics Committee Bern). All residents responsible for in-hospital admissions in the participating study centers will be asked to participate in our study and to provide written informed consent. For the main endpoint of the study (code status), the Ethics Committees agreed, that patient level informed consent is not necessary, due to the cluster randomization design. However, all patients will be asked to give written informed consent for completion of questionnaires related to secondary endpoints.

2.2.5. Study flow

Code status discussions are usually conducted by the treating residents on the ward. Residents that agree to participate will be randomized into the intervention or control group with a 1:1 allocation (see Figure 1). Randomization will be done by a pre-specified, computer-generated, web-based randomization scheme (block randomization with variables sizes of 4 to 6). The randomization will be stratified for the trial site. The CTU Basel is responsible for the integrity of the randomization procedures and randomization will be electronically integrated in the SecuTrial® website, where also all data collection for this trial is done.

The study will be conducted for admissions from Monday to Friday. Admission on medical wards normally takes place between 8:00 am and 4:00 pm. Residents that participate in our study will report all new admissions to our study team. Additionally, we will check the hospital’s database and the hospital’s bed managers will provide us with a list of planned hospitalizations one day in advance. A member of our study team will then check whether the new patient is eligible for trial inclusion.

Figure 1. Study flow

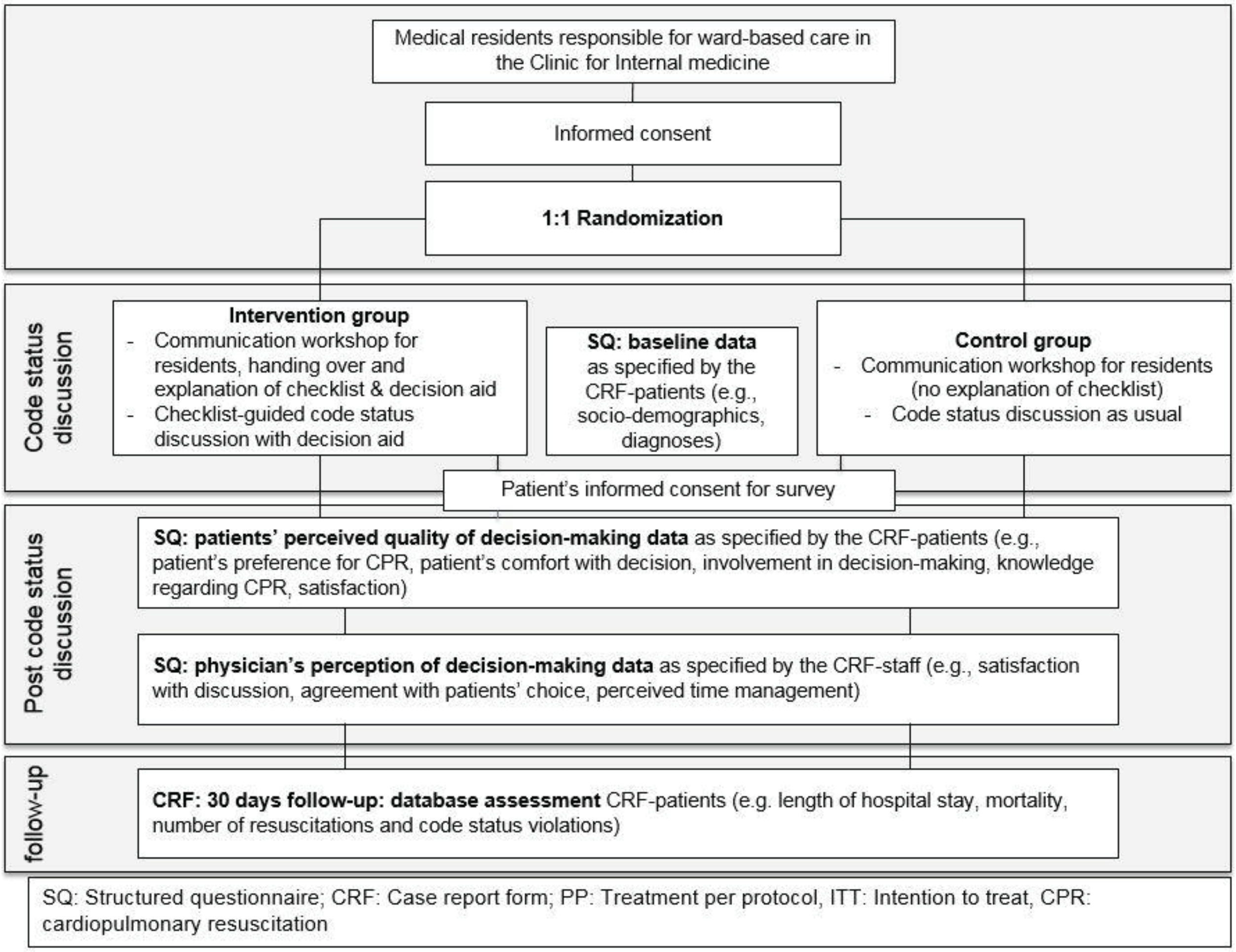

Baseline data will be entered into a centralized web-based study software (SecuTrial®) provided by the Clinical Trial Unit (CTU). The following baseline-data will be recorded for each patient:

- Socio-demographics (e.g., age, gender, socio-economic status, religion,)
- Health-related quality of life defined by EQ-5D (EuroQol)[46]
- Medical situation (i.e., complete list of main diagnosis and comorbidities)

To enable blinding of patients and physicians regarding the intervention and main endpoint of our trial, we will not share any detailed information, but ask for their participation in a trial comparing two different communication strategies on the quality of code status discussions. Yet, blinding of the study coordinator is not feasible. After code status discussion, a member of our research team will interview patients and residents separately. Using a structured questionnaire, patient outcomes and perceived quality of care as defined below (2.2.8. primary and secondary endpoints) will be assessed. In addition, the physician's perception of the code status discussion will be examined using a structured questionnaire.

2.2.6. Interventions

For the purpose of standardization and in line with current practice, in both groups code status discussions will take place upon hospitalization. Residents allocated to the intervention group will participate in a communication workshop, where they will receive detailed instructions about the shared decision-making checklist including specific communication teachings. Additionally, residents will be asked to use a

Figure 2. CLEAR Checklist

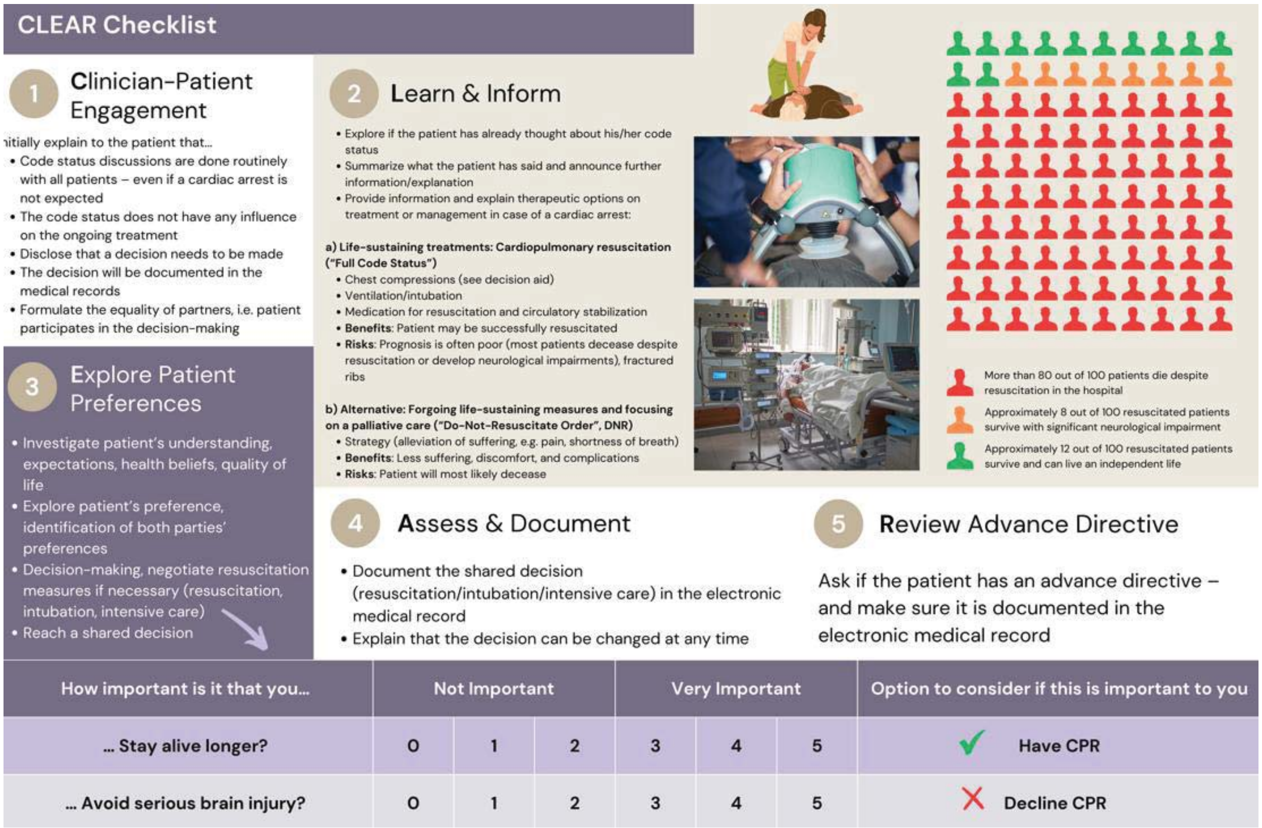

structured checklist for shared decision-making and a decision aid (see Figure 2), depicting a simulated CPR and a mechanically ventilated patient on an intensive care unit, as well as addressing prognostic data and outcomes of resuscitation. The decision aid from our study is based on previously published data and partially adapted from other decision aids that are registered in the Ottawa decision aid inventory[47]. To create the checklist, we grouped aspects of shared decision-making into five categories, from which we generated the acronym "CLEAR" (**C**linician-Patient Engagement, **L**earn and **I**nform, **E**xplore Patient Preferences, **A**ssess and Document, **R**eview Advance Directives). During the one-hour workshop, which was previously pilot-tested, residents randomized to the intervention group receive a teaching about shared decision-making including use of the checklist and decision aid, and will have to conduct a code status discussion with a simulated patient using the checklist and the decision aid. A member of our study team then will provide critical feedback how to facilitate the use of checklist and decision aid.

To accomplish fidelity to the intervention, the member of our study team who conducted the workshop and is not involved in the patient surveys, will accompany the resident for at least three code status discussions. Each time, we will provide feedback to residents regarding the discussion and – if necessary –a short rehearsal of single components of the training. To reduce bias, medical residents in the control group will also receive information about the importance of code status discussions and a general communication training (e.g., information structuring, responding to emotions) but no teaching regarding shared decision-making and no checklist or decision aid will be used. Communication workshops will be conducted before the resident will start conducting code status discussions, by members of our study team that will not have further contact with physicians and patients to improve blinding to group allocation.

2.2.7. Patient involvement

A total of 25 patients hospitalized on the medical wards of the University Hospital of Basel, as well as 15 healthcare providers (physicians and nurses) with experience in end-of-life care and emergency/intensive care medicine were involved in the design of the study and intervention as well as the review of the study protocol. To design the trial, patients and healthcare providers helped us in prioritization and selection of outcomes. Patients were asked for priority focus of this study. To design the intervention, input was sought from patients and healthcare providers. Especially, the decision aid was shown to patients to ascertain whether the depicted medical images or information would raise discomfort. Additionally, the decision aid and checklist were adapted according to inputs from clinical experts.

2.2.8. Primary and secondary endpoints

This trial is designed to investigate whether checklist-guided shared decision-making influences patients` decision regarding DNR code status, and at the same time improves decision-making quality. The primary outcome is thus the frequency of DNR code status among patients per resident (i.e., patients choosing that CPR and intubation measures should not be performed in case of acute deterioration). To ascertain the final code status decision, we will look at the code status documented in the medical chart. Code status decisions will be defined as “yes” (preference for CPR) and “no” (DNR).

As patients in our previous survey also prioritized the ability to make a reasonable decision, we have chosen patients’ decisional conflict and comfort with the chosen code status as the key secondary endpoint, assessed through the validated German translation of the Decision Conflict Scale. The Decisional Conflict Scale is a validated questionnaire widely known to measure patients’ uncertainty in choosing between treatment options, modifiable factors associated with this uncertainty as well as perceived effective decision-making. Further outcomes of interest mentioned by patients and healthcare providers were included as secondary outcomes and are detailed below.

Additional secondary endpoints are defined as follows.

1. Patient-associated outcomes

a) Patients' knowledge about resuscitation measures and expected outcome of an in-hospital cardiac arrest. Knowledge will be defined as a score ascertained by a knowledge questionnaire that was adapted from previous studies [48, 49] and translated into German. The questionnaire of this study contains 6 questions regarding CPR and its prognostic outcome. Every correct answer given by the patient will add up to a score (between 0 and 6). State of not knowing the answer will be rated as a wrong answer.
b) Patients' involvement in the decision-making process will be assessed through the PEF-FB-9, a validated German translation of the SDM-q-9 questionnaire[50]. The SDM-q-9 questionnaire is a well-established tool to measure quality of shared decision-making
c) Patients' concerns and fears brought up by the resuscitation discussion: Specifically, we will assess the following dimensions: (1) disturbance caused by the discussion, (2) fear of suffering from an actual cardiac arrest, (3) fear of suffering from a life-threatening disease (4) perceived feeling of being put under pressure during discussion; each of them rated on a VAS (0-10).
d) Patients' satisfaction and perceived overall quality of the code status discussions: Specifically, we will focus on the following dimensions, each rated on a VAS (0-10): (1) overall satisfaction with resuscitation discussion; (2) perceived transparency of the discussion; (3) comprehensibility of the medical information regarding a resuscitation process and measures; (4) how well resuscitation options were elucidated

2. Physician- associated outcomes

a) Perception of the physician with the resuscitation discussion: Specifically, perceptions will be assessed for both study groups separately in regard to the following dimensions: (1) satisfaction with resuscitation discussion and self-confidence during the discussion, each rated on a VAS (0-10); (2) perceived time management of the discussion, rated on a VAS (0-10)
b) Surprise question to identify patients at risk of death within the next 12 months

3. Documented results in Database after 30 days

Database assessment after 30 days: Specifically, we are interested in (1) length of stay; (2) number of ICU admissions; (3) number of re-admissions to the hospital; (4) 30d mortality

2.3.9 Statistical approach

The primary analysis population is the full analysis set, which, following intention-to treat (ITT) principles, includes all randomized patients.

We will use cluster specific methods because residents rather than patients are randomized, and we expected that variance in how patients are managed would be partly explained by the resident. We will further account for the level of the participating center (hospital), as DNR discussions may vary according to study center. For each of the outcome measures we will test the null hypothesis that the likelihood of the outcome is the same in both study groups after accounting for some baseline characteristics (i.e., age, gender, main medical diagnosis). We will use hierarchical logistic regression to test for an effect of the intervention on any binary outcome measures and linear regression for any continuous outcome measure.

All analyses will be conducted using Stata All analyses were performed using STATA 15.1 (Stata Corp, College Station, TX). For all analyses, we accept a type I error rate of alpha = 5%.

The design of our study and statistical approach was developed in close collaboration with statisticians and epidemiologists of the Clinical Trial Unit (CTU) of the University Hospital Basel.

2.3.10 Power and sample size considerations

For sample size calculation, we consider the intra-cluster correlation coefficient, the number of events, the expected effect, and the power of the study. Based on pilot data in this patient population, we expect that 30% of patients in the usual care control will have a DNR code status, which will increase to 45% in the intervention group. We plan to include 174 residents (cluster) with a mean of 5 eligible patients per week over a 3-week period (total of 15 patients per cluster) in the five participating centers over a total study time of 2 years. Based on these assumptions, we will include 2610 patients among 174 residents, which will give this study an 80% power at a 0.05 alpha error with an inter-cluster correlation of 0.5. Because the inter-cluster correlation in this setting is unknown, we used a conservative estimate above, but plan to possibly adjust the sample size based on a blinded interim analysis after data of 30 residents (cluster) is collected. In this interim analysis, we estimate the intraclass correlation of code status decisions, i.e., how much the code status decisions are cluster-dependent. Based on this estimation of intraclass correlation, we will adjust the total required sample size.

2.3.11 Recruitment and recruitment feasibility

To achieve our calculated sample size of 174 residents, each included for a 3-week period, in the five centers, we plan to include 2-3 residents in parallel (totaling to 35-53 residents per year per center). All centers have between 20-60 residents per year, which stay at a center for 1-3 years. Thus, due to rotation we expect to have between 30 and 90 eligible residents per center per year. We will include centers stepwise. In case, one or more centers fall short of recruitment, we plan to increase the time for recruitment in the other centers until our sample size goals are reached.

Strategies to achieve the estimated inclusion rate will include:

(1) A close monitoring of recruitment rates with monthly visits of study sites by one member of our study team will help to facilitate recruitment and remove potential barriers.
(2) Each center will receive a contact list with the mobile numbers of the study coordinator and the principal investigator to enable support and clarification in situations, in which local investigators might be unsure, whether to recruit a patient.
(3) Monthly newsletters and alerts will be sent to participating centers indicating the actual progress of the study.
(4) Monthly investigator meetings will be held to exchange experiences, discuss processes and problems to guarantee an uniform approach between all participating study sites.
(5) Quarterly information meetings will be held in all participating hospitals to inform healthcare providers about the ongoing study.

We have successfully used these approaches in a currently recruiting multicenter randomized trial supported by the Swiss National Foundation (SNF 10531C_182422) investigating the effects of ward-rounds on patients' perception of quality of care (http://clinicaltrials.gov/ct2/show/NCT03210987).

To make a reasonable estimate of included residents and eventually recruited patients in our study, we chose a stepwise approach: First, we obtained the total number of residents working on ward-based medicine responsible for admissions. Second, the annual report of each participating center provides us the number of hospitalized patients that enable us to make a sound estimation of potentially eligible patients. All participating centers currently have between 4220 and 8000 hospitalized patients annually.

2.3.12 Potential limitations

We expect heterogeneity regarding our patient population. However, we decided against excluding any patient based on the type of illness and comorbidity in order to obtain a representative population of medical patient in Swiss hospitals.

2.4 Parallel study: Code status discussions in patients with futility regarding CPR

Cardiopulmonary resuscitation (CPR) is a medical invasive treatment in case of a cardiac arrest. The Swiss Academy for Medical Sciences recommends that healthcare providers start resuscitation attempts if there is a chance for the patient to survive a cardiac arrest without severe neurologic deficits, unless a "do not resuscitate" (DNR) code status is present. In contrast, for patients with terminal illness CPR may not always be appropriate. Outcome after in-hospital cardiac arrest is generally poor with a survival rate of approximately 20% and many survivors subsequently suffer from permanent brain damage or disability[23, 24]. Certain pre-existing medical conditions such as cancer, renal failure, sepsis or pulmonary diseases are associated with even worse outcomes. This raises the question of futility regarding resuscitation measures[51, 52]. In this context, medical futility may be defined as a judgment that further medical treatment of a patient would have no useful result, or that a medical treatment whose success is possible although reasoning and experience suggest that it is highly improbable.

In some countries including Switzerland, physicians are allowed to put a unilateral DNR in place in case resuscitation measures are judged as futile[53, 54]. In line with this, the American Heart Association Guidelines state that withholding CPR is ethically justified if functional survival of patients is highly unlikely[55]. However, there is no universally accepted method to assess futility. In clinical practice, evaluation of futility is mainly based on the clinician's judgement and patient characteristics such as age and comorbidities.

Recently Ebell et al developed the GO-FAR score, a clinical risk score to predict the estimated likelihood of neurologically intact survival of patients after in-hospital resuscitation[41]. The authors developed a multivariate prediction model based on 51`240 patients who experienced in-hospital cardiac arrest and calculated cut-off scores indicating likelihood of functional survival. The two lowest categories represent very low (<1%) and low (1-3%) likelihood of survival. It has therefore been suggested that these patients should be considered futile regarding CPR attempts [42]. Another tool to assess whether patients are at increased risk for poor outcome is the Clinical Frailty Scale[43]. This 9-category scale combines cognitive and physical aspects of frailty such as comorbidity, cognitive impairment and disability. Research has shown that the scale, which is increasingly used in routine screening of older patients, predicts short-term mortality[44, 45]. However, the prevalence of futility in hospitalized patients upon admission assessed through the GO-FAR score and the Clinical Frailty Scale has not been systematically investigated.

According to the literature, futility may be defined as a GO-FAR score ≥ 14 or patients in categories 7-9 in the Clinical frailty scale. In 2012, a British multi-center cohort study assessed medical charts of cardiac arrest patients, in which resuscitation attempts had been performed[26]. In more than 3 of 4 patients the code status was unknown and 67% of patients that were resuscitated, had an underlying pre-existing fatal disease. An independent post-hoc assessment of all cases found that a DNR status would have been appropriate in 85% of cases, because resuscitation attempts were judged to be futile and thus CPR had little chance to be beneficial, but high risk to prolong the dying process[56].

However, informing a patient that resuscitation measures in case of a cardiac arrest are judged as futile and setting a DNR status is complex and challenging. Due to their limited experience in code status discussion, this might be especially true for unexperienced medical residents.

In this ancillary project we aim to examine, whether a communication workshop and a checklist for communication about futility is associated with a higher rate of DNR code status in patients who are considered futile according to either the Go-Far Score or the Clinical Frailty Scale.

Interventions of the parallel project

Patients, in whom resuscitation attempts are considered futile defined as a Go-Far score ≥ 14 or patients in categories 7-9 in the Clinical frailty scale, will be included in this ancillary project. Similar to the main project, code status discussions will take place upon admission of patients. Residents allocated to the intervention

group will participate in the above mentioned communication workshop for the main project. During the workshops, residents will also receive detailed instructions about communication strategies in patients with futility. Additionally, residents in the intervention group will be given a checklist for communication of futility (Appendix). To reduce bias, medical residents in the control group will also receive information about the importance of code status discussions and general communication training, but no teaching regarding communication of futility.

Outcomes in parallel project

The parallel project is designed to investigate whether checklist-guided communication of futility increases the rate of DNR code status. The primary outcome is thus the frequency of DNR code status among patients per resident.

Secondary outcomes are defined as follows:

1. Patient-associated outcomes

a) Patients' trust in the treating physician assessed through the validated German translation of the Trust in Physician Scale[57].
b) Patients' psychological burden assessed by the validated German translation of the Hospital Anxiety and Depression Scale (HADS)[58].
c) Patients' concerns and fears assessed by the validated German translation of the State-Trait Anxiety Inventory[59]
d) Patients' concerns and fears brought up by the resuscitation discussion: Specifically, we will assess the following dimensions: (1) disturbance caused by the discussion, (2) fear of suffering from an actual cardiac arrest, (3) fear of suffering from a life-threatening disease (4) perceived feeling of being put under pressure during discussion; each of them rated on a VAS (0-10).
e) Patients' satisfaction and perceived overall quality of the code status discussions: Specifically, we will focus on the following dimensions, each rated on a VAS (0-10): (1) overall satisfaction with resuscitation discussion; (2) perceived transparency of the discussion; (3) comprehensibility of the medical information regarding a resuscitation process and measures; (4) clarity of the medical information regarding a resuscitation process and measures; (5) how well patients' questions were answered; (6) agreement with the decision of putting a DNR code status in place; (7) perceived competence of the medical team; (8) perceived listening skills of the physician;

Physician-associated outcomes

a) Physicians' perception of the resuscitation discussion: Specifically, perceptions will be assessed for both study groups separately in regard to the following dimensions; each rated on a VAS (0-10): (1) satisfaction with resuscitation discussion and self-confidence during the discussion; (2) perceived time management of the discussion, (3) physicians' perceived understanding and concerns of patients, (4) physicians' perception of patient indecisiveness and agreement
b) Surprise question to identify patients at risk of death within the next 12 months

After 30 days we will assess length of hospital stay, ICU admissions, if any changes in code status occurred, in-hospital resuscitations, code status violations and in-hospital deaths through medical chart review.

2.5 Relevance and impact

Proactive involvement of patients into the decision process is an important part of patient-centered care. Still, few randomized controlled studies have focused on the best approach to conduct codes status discussions. Herein, we propose the first large-scale multicenter trial that will give important, new insights on the effect of checklist-guided shared decision-making on patients' choice for DNR and decisional comfort with their choice as well as on other patient- and healthcare-relevant outcomes. Considering different dimensions of quality of care such as patients' understanding will help to realize a more patient-centered medicine by enabling well-informed patients to take part in a shared decision-making process.

3 Bibliography

1. Committee on Quality of Health Care in America, Medicine Io: **Crossing the Quality Chasm: A New Health System for the 21st Center.** In. Washington, DC: National Academy Press; 2001.
2. **Good care of the dying patient. Council on Scientific Affairs, American Medical Association**. *JAMA* 1996, **275**(6):474-478.
3. Lo B, Snyder L: **Care at the end of life: guiding practice where there are no easy answers**. *Ann Intern Med* 1999, **130**(9):772-774.
4. Lynn J: **Measuring quality of care at the end of life: a statement of principles**. *J Am Geriatr Soc* 1997, **45**(4):526-527.
5. Back AL, Anderson WG, Bunch L, Marr LA, Wallace JA, Yang HB, Arnold RM: **Communication about cancer near the end of life**. *Cancer* 2008, **113**(7 Suppl):1897-1910.
6. Lo B, McLeod GA, Saika G: **Patient attitudes to discussing life-sustaining treatment**. *Arch Intern Med* 1986, **146**(8):1613-1615.
7. Nicolasora N, Pannala R, Mountantonakis S, Shanmugam B, DeGirolamo A, Amoateng-Adjepong Y, Manthous CA: **If asked, hospitalized patients will choose whether to receive life-sustaining therapies.** *J Hosp Med* 2006, **1**(3):161-167.
8. Elkin EB, Kim SH, Casper ES, Kissane DW, Schrag D: **Desire for information and involvement in treatment decisions: elderly cancer patients' preferences and their physicians' perceptions**. *J Clin Oncol* 2007, **25**(33):5275-5280.
9. Tulsky JA, Chesney MA, Lo B: **How do medical residents discuss resuscitation with patients?** *J Gen Intern Med* 1995, **10**(8):436-442.
10. Einstein DJ, Einstein KL, Mathew P: **Dying for Advice: Code Status Discussions between Resident Physicians and Patients with Advanced Cancer--A National Survey**. *J Palliat Med* 2015, **18**(6):535-541.
11. Anderson WG, Chase R, Pantilat SZ, Tulsky JA, Auerbach AD: **Code status discussions between attending hospitalist physicians and medical patients at hospital admission**. *J Gen Intern Med* 2011, **26**(4):359-366.
12. Marco CA, Larkin GL: **Cardiopulmonary resuscitation: knowledge and opinions among the U.S. general public. State of the science-fiction**. *Resuscitation* 2008, **79**(3):490-498.
13. Diem SJ, Lantos JD, Tulsky JA: **Cardiopulmonary resuscitation on television. Miracles and misinformation**. *N Engl J Med* 1996, **334**(24):1578-1582.
14. Tulsky JA, Fischer GS, Rose MR, Arnold RM: **Opening the black box: how do physicians communicate about advance directives?** *Ann Intern Med* 1998, **129**(6):441-449.
15. Covinsky KE, Fuller JD, Yaffe K, Johnston CB, Hamel MB, Lynn J, Teno JM, Phillips RS: **Communication and decision-making in seriously ill patients: findings of the SUPPORT project. The Study to Understand Prognoses and Preferences for Outcomes and Risks of Treatments**. *J Am Geriatr Soc* 2000, **48**(5 Suppl):S187-193.
16. Billings ME, Curtis JR, Engelberg RA: **Medicine residents' self-perceived competence in end-of-life care**. *Acad Med* 2009, **84**(11):1533-1539.
17. Deep KS, Griffith CH, Wilson JF: **Communication and decision making about life-sustaining treatment: examining the experiences of resident physicians and seriously-ill hospitalized patients**. *J Gen Intern Med* 2008, **23**(11):1877-1882.
18. Momen NC, Barclay SI: **Addressing 'the elephant on the table': barriers to end of life care conversations in heart failure - a literature review and narrative synthesis**. *Curr Opin Support Palliat Care* 2011, **5**(4):312-316.
19. Knauft E, Nielsen EL, Engelberg RA, Patrick DL, Curtis JR: **Barriers and facilitators to end-of-life care communication for patients with COPD**. *Chest* 2005, **127**(6):2188-2196.

20. Visser M, Deliens L, Houttekier D: **Physician-related barriers to communication and patient- and family-centred decision-making towards the end of life in intensive care: a systematic review**. *Crit Care* 2014, **18**(6):604.
21. Rocker G, Cook D, Sjokvist P, Weaver B, Finfer S, McDonald E, Marshall J, Kirby A, Levy M, Dodek P *et al*: **Clinician predictions of intensive care unit mortality**. *Crit Care Med* 2004, **32**(5):1149-1154.
22. Curtis JR, Vincent JL: **Ethics and end-of-life care for adults in the intensive care unit**. *Lancet* 2010, **376**(9749):1347-1353.
23. Perkins GD, Cooke MW: **Variability in cardiac arrest survival: the NHS Ambulance Service Quality Indicators**. *Emerg Med J* 2012, **29**(1):3-5.
24. Meaney PA, Nadkarni VM, Kern KB, Indik JH, Halperin HR, Berg RA: **Rhythms and outcomes of adult in-hospital cardiac arrest**. *Crit Care Med* 2010, **38**(1):101-108.
25. Girotra S, Chan PS: **Trends in survival after in-hospital cardiac arrest**. *N Engl J Med* 2013, **368**(7):680-681.
26. **Time to intervene? A review of patients who underwent cardiopulmonary resuscitation as a result of an in-hospital cardiorespiratory arrest. A report by the National Confidential Enquiry into Patient Outcome and Death (2012)**
27. El-Jawahri A, Lau-Min K, Nipp RD, Greer JA, Traeger LN, Moran SM, D'Arpino SM, Hochberg EP, Jackson VA, Cashavelly BJ *et al*: **Processes of code status transitions in hospitalized patients with advanced cancer**. *Cancer* 2017, **123**(24):4895-4902.
28. Szmuilowicz E, Neely KJ, Sharma RK, Cohen ER, McGaghie WC, Wayne DB: **Improving residents' code status discussion skills: a randomized trial**. *J Palliat Med* 2012, **15**(7):768-774.
29. Seoane L, Bourgeois DA, Blais CM, Rome RB, Luminais HH, Taylor DE: **Teaching palliative care in the intensive care unit: how to break the news**. *Ochsner J* 2012, **12**(4):312-317.
30. Han PK, Keranen LB, Lescisin DA, Arnold RM: **The palliative care clinical evaluation exercise (CEX): an experience-based intervention for teaching end-of-life communication skills**. *Acad Med* 2005, **80**(7):669-676.
31. Amro OW, Ramasamy M, Strom JA, Weiner DE, Jaber BL: **Nephrologist-Facilitated Advance Care Planning for Hemodialysis Patients: A Quality Improvement Project**. *Am J Kidney Dis* 2016, **68**(1):103-109.
32. Andereck WS, McGaughey JW, Schneiderman LJ, Jonsen AR: **Seeking to reduce nonbeneficial treatment in the ICU: an exploratory trial of proactive ethics intervention***. *Crit Care Med* 2014, **42**(4):824-830.
33. Schneiderman LJ, Gilmer T, Teetzel HD: **Impact of ethics consultations in the intensive care setting: a randomized, controlled trial**. *Crit Care Med* 2000, **28**(12):3920-3924.
34. McCannon JB, O'Donnell WJ, Thompson BT, El-Jawahri A, Chang Y, Ananian L, Bajwa EK, Currier PF, Parikh M, Temel JS *et al*: **Augmenting communication and decision making in the intensive care unit with a cardiopulmonary resuscitation video decision support tool: a temporal intervention study**. *J Palliat Med* 2012, **15**(12):1382-1387.
35. El-Jawahri A, Mitchell SL, Paasche-Orlow MK, Temel JS, Jackson VA, Rutledge RR, Parikh M, Davis AD, Gillick MR, Barry MJ *et al*: **A Randomized Controlled Trial of a CPR and Intubation Video Decision Support Tool for Hospitalized Patients**. *J Gen Intern Med* 2015, **30**(8):1071-1080.
36. Volandes AE, Levin TT, Slovin S, Carvajal RD, O'Reilly EM, Keohan ML, Theodoulou M, Dickler M, Gerecitano JF, Morris M *et al*: **Augmenting advance care planning in poor prognosis cancer with a video decision aid: a preintervention-postintervention study**. *Cancer* 2012, **118**(17):4331-4338.
37. Volandes AE, Paasche-Orlow MK, Mitchell SL, El-Jawahri A, Davis AD, Barry MJ, Hartshorn KL, Jackson VA, Gillick MR, Walker-Corkery ES *et al*: **Randomized controlled trial of a video decision support tool for cardiopulmonary resuscitation decision making in advanced cancer**. *J Clin Oncol* 2013, **31**(3):380-386.
38. Overdyk FJ, Dowling O, Marino J, Qiu J, Chien HL, Erslon M, Morrison N, Harrison B, Dahan A, Gan TJ: **Association of Opioids and Sedatives with Increased Risk of In-Hospital**

Cardiopulmonary Arrest from an Administrative Database. *PLoS One* 2016, **11**(2):e0150214.
39. **A controlled trial to improve care for seriously ill hospitalized patients. The study to understand prognoses and preferences for outcomes and risks of treatments (SUPPORT). The SUPPORT Principal Investigators**. *JAMA* 1995, **274**(20):1591-1598.
40. Hess EP, Hollander JE, Schaffer JT, Kline JA, Torres CA, Diercks DB, Jones R, Owen KP, Meisel ZF, Demers M *et al*: **Shared decision making in patients with low risk chest pain: prospective randomized pragmatic trial**. *BMJ* 2016, **355**:i6165.
41. Ebell MH, Jang W, Shen Y, Geocadin RG, Get With the Guidelines-Resuscitation I: **Development and validation of the Good Outcome Following Attempted Resuscitation (GO-FAR) score to predict neurologically intact survival after in-hospital cardiopulmonary resuscitation**. *JAMA Intern Med* 2013, **173**(20):1872-1878.
42. Schneiderman LJ, Jecker NS, Jonsen AR: **Medical futility: its meaning and ethical implications**. *Ann Intern Med* 1990, **112**(12):949-954.
43. Rockwood K, Song X, MacKnight C, Bergman H, Hogan DB, McDowell I, Mitnitski A: **A global clinical measure of fitness and frailty in elderly people**. *CMAJ* 2005, **173**(5):489-495.
44. Gregorevic KJ, Hubbard RE, Lim WK, Katz B: **The clinical frailty scale predicts functional decline and mortality when used by junior medical staff: a prospective cohort study**. *BMC Geriatr* 2016, **16**:117.
45. Chua XY, Toh S, Wei K, Teo N, Tang T, Wee SL: **Evaluation of clinical frailty screening in geriatric acute care**. *J Eval Clin Pract* 2019.
46. EuroQol G: **EuroQol--a new facility for the measurement of health-related quality of life**. *Health policy (Amsterdam, Netherlands)* 1990, **16**(3):199-208.
47. Krones T, Budilivschi A, Karzig I, Otto T, Valeri F, Biller-Andorno N, Mitchell C, Loupatatzis B: **Advance care planning for the severely ill in the hospital: a randomized trial**. *BMJ Support Palliat Care* 2019.
48. Kerridge IH, Pearson SA, Rolfe IE, Lowe M, McPhee JR: **Impact of written information on knowledge and preferences for cardiopulmonary resuscitation**. *Med J Aust* 1999, **171**(5):239-242.
49. Stein RA, Sharpe L, Bell ML, Boyle FM, Dunn SM, Clarke SJ: **Randomized controlled trial of a structured intervention to facilitate end-of-life decision making in patients with advanced cancer**. *J Clin Oncol* 2013, **31**(27):3403-3410.
50. Kriston L, Scholl I, Holzel L, Simon D, Loh A, Harter M: **The 9-item Shared Decision Making Questionnaire (SDM-Q-9). Development and psychometric properties in a primary care sample**. *Patient education and counseling* 2010, **80**(1):94-99.
51. Ebell MH, Afonso AM: **Pre-arrest predictors of failure to survive after in-hospital cardiopulmonary resuscitation: a meta-analysis**. *Fam Pract* 2011, **28**(5):505-515.
52. de Vos R, de Haes HC, Koster RW, de Haan RJ: **Quality of survival after cardiopulmonary resuscitation**. *Arch Intern Med* 1999, **159**(3):249-254.
53. Miceli M: **Bioethics in Practice: Unilateral Do-Not-Resuscitate Orders**. *Ochsner J* 2016, **16**(2):111-112.
54. van Delden JJ, Lofmark R, Deliens L, Bosshard G, Norup M, Cecioni R, van der Heide A, Consortium E: **Do-not-resuscitate decisions in six European countries**. *Crit Care Med* 2006, **34**(6):1686-1690.
55. Mancini ME, Diekema DS, Hoadley TA, Kadlec KD, Leveille MH, McGowan JE, Munkwitz MM, Panchal AR, Sayre MR, Sinz EH: **Part 3: Ethical Issues: 2015 American Heart Association Guidelines Update for Cardiopulmonary Resuscitation and Emergency Cardiovascular Care**. *Circulation* 2015, **132**(18 Suppl 2):S383-396.
56. **Guidelines for the appropriate use of do-not-resuscitate orders. Council on Ethical and Judicial Affairs, American Medical Association**. *JAMA* 1991, **265**(14):1868-1871.
57. Anderson LA, Dedrick RF: **Development of the Trust in Physician scale: a measure to assess interpersonal trust in patient-physician relationships**. *Psychol Rep* 1990, **67**(3 Pt 2):1091-1100.

58. Zigmond AS, Snaith RP: **The hospital anxiety and depression scale**. *Acta Psychiatr Scand* 1983, **67**(6):361-370.
59. Laux L, Glanzmann P, Schaffner P, Spielberger CD: **Das State-Trait-Angstinventar [The state-trait anxiety inventory]**. *Hogrefe, Göttingen (in German)* 1981.
