# Supplementary material for: Checklist-Guided Code Status Discussions in Patients for Whom Cardiopulmonary Resuscitation Is Considered Futile: An Analysis of a Randomized Clinical Trial
Source: JAMA Netw Open. 2025 Sep 25;8(9):e2533638. doi: 10.1001/jamanetworkopen.2025.33638 (PMC12464790; doi:10.1001/jamanetworkopen.2025.33638)
Supplement: Supplement 3. — Data Sharing Statement [file jamanetwopen-e2533638-s003.pdf]

Data Sharing Statement

Arpagaus. Checklist-Guided Code Status Discussions in Patients for Whom Cardiopulmonary Resuscitation Is Considered Futile. *JAMA Netw Open*. Published September 25, 2025. doi:10.1001/jamanetworkopen.2025.33638

Data
Additional Information: ClinicalTrials.gov; https://clinicaltrials.gov/ct2/show/NCT03872154, NCT03872154
Data available: No

Additional Information
Explanation for why data not available: The data that support the findings of this study are available from the corresponding author upon reasonable request.
